# Built out cities? A new approach to measuring land use regulation

**DOI:** 10.1016/j.jhe.2024.101982

**Published:** 2024-02-09

**Authors:** Paavo Monkkonen, Michael Manville, Michael Lens

**Affiliations:** University of California, Los Angeles, United States

**Keywords:** R15, R31, R38, R52, Land use regulation, Measurement, Housing production

## Abstract

We introduce a new way to measure the stringency of housing regulation. Rather than a standard regulatory index or a single aspect of regulation like Floor Area Ratio, we draw on cities’ self-reported estimates of their total zoned capacity for new housing. This measure, available to us as a result of state legislation in California, offers a more accurate way to assess local antipathy toward new housing, and also offers a window into how zoning interacts with existing buildout. We show, in regressions analyzing new housing permitting, that our measure has associations with new supply that are as large or larger than conventional, survey-based indexes of land use regulation. Moreover, unbuilt zoning capacity interacts with rent to predict housing production in ways conventional measures do not. Specifically, interacting our measure with rent captures the interplay of regulation and demand: modest deregulation in high-demand cities is associated with substantially more housing production than substantial deregulation in low-demand cities. These findings offer a more comprehensive explanation for the historically low levels of housing production in high cost metros.

## 1. Introduction

This article examines how local regulations restrict housing supply, using California as a case study. By now most researchers agree that regulation plays some role in high housing prices (Kahn et al., 2010; Kok et al., 2014; Jackson, 2018). But regulation is both a broad term and a black box. A wide variety of regulations exist, and while the literature has found a strong correlation between regulatory stringency and housing prices, these findings are accompanied by two caveats: Regulation is hard to measure consistently, and the mechanisms through which it increases price remain opaque.

The opacity arises for two related reasons. First is the sheer variety of regulations, and regulatory decisions, that localities can choose from. Cities can use different regulations to arrive at the same outcome of low housing production. One local government might have large minimum lot sizes, another high minimum parking requirements, while a third might look lenient on paper but then review each project slowly and use its discretionary authority to discourage or reject applications for entitlement. These three cities could be equally adept at blocking development, but analysts correlating any *given* regulation with housing supply and price across these cities might find only weak relationships, even if they measured that regulation perfectly.

The second reason, which flows from the first, is that individual regulations might be interchangeable proxies for a larger phenomenon, which is an underlying and hard-to-observe political antipathy to new housing. A high minimum lot size can be a legitimately binding constraint. But suppose a city with such a requirement was forced to remove it. Would this city just welcome more housing? Or would it increase its parking requirements, reduce its height limits, or start slow-walking permit applications? Regulations are adopted for a reason, and may therefore be more symptom than source.

Our primary contribution in this paper is a novel way to measure that source—the city’s underlying sentiment toward housing development. Our measure is the city’s subjective (but quantified) judgment of how close it is to its own buildout point, or what we call its “unbuilt capacity.” This measure is available in California as a result of the state’s Housing Element law. The law requires all local jurisdictions in California to regularly estimate their unbuilt capacity and report it to the state. This requirement is compulsory, municipal governments take it seriously, and—crucially—because the reporting has real-world consequences, cities are strategic when they do it, and many try to keep their estimates low.

Strategic behavior is possible—and likely—because buildout estimates are fundamentally subjective. The estimates often rest on objective measures (e.g., the availability of vacant land, and units allowed under existing zoning), but all of California’s cities could physically hold more housing *if* they decided to allow it. Allowing housing is primarily a political decision. When cities report their ability to hold new housing, therefore, they reveal an overall tolerance for development—a tolerance that is reflected in different forms of regulation across both places and time.

The subjectivity of buildout is a crucial assumption, and one we revisit elsewhere the paper. For now, though, here is an example. In 1960 the total zoned capacity of the city of Los Angeles suggested it could hold roughly 10 million people. By 1990 the zoned capacity had fallen to where the city could hold about 4.5 million; it remains at this point today (Morrow, 2013). What accounts for this 55 % decline? Los Angeles did not add mountains or water between 1960 and 1990. It did not lose land area. Structural engineers did not determine that buildings in Los Angeles could not be as tall as was once thought (if anything the opposite was true; improvements in seismic safety made taller structures more feasible). What changed was residents’ attitudes toward development—prompted, in part, by development itself (Morrow, 2013; Whittemore, 2011). These changed attitudes came to be reflected, in different ways, in the city’s regulations. Los Angeles had once determined that it could hold twice as much housing as it says it can now. It could return to that higher determination *if it wanted to*.

Unbuilt capacity is not a land use regulation. It is a number that estimates the underlying political attitude that land use regulations make manifest. In this way, it accomplishes what many indices of regulation attempt. We show in this paper that in simple models of new housing supply (as measured by building permits), unbuilt capacity performs comparably to a conventional index of regulation. We also show, however, that unbuilt capacity has an explanatory power the conventional index lacks: it offers a better window into the relationship between regulation and demand. When we interact a lagged rent variable with conventional metrics of regulation, the interaction term is statistically insignificant. When we do the same with unbuilt capacity, in contrast, the results are significant both economically and statistically. The results suggest that unbuilt capacity is strongly associated with permitting in high rent cities, but *not* in low rent cities. Specifically, we see that when demand is low, even dramatic changes in regulatory stringency (as measured by buildout) yield relatively little new housing, while when demand is high, even modest deregulation is associated with more permitting.

Our results are not causal. However, because our dependent variable is supply rather than price, any endogeneity present in our regressions likely biases our coefficients toward zero. Regulation can reduce supply (our hypothesized outcome), but new housing supply can also induce regulation (a simultaneity threat that works against our hypothesis). Because this bias is unaddressed in our regressions, our results may plausibly be underestimates.

Our findings about the interaction of demand and regulation are at once economically unsurprising and counterintuitive to policymakers. They are economically unsurprising because development is more likely to occur in higher-demand (higher-priced) neighborhoods, but development also creates its own opposition: a political constituency that wants to defend a low density status quo. When these constituencies gain sufficient power, they enact regulations that slow or prevent development (Ellickson 2022; Fischel 2009). The net result is a city where housing construction is less likely to occur than it would have been absent regulation, but *more* likely to occur than in other places where demand is lower. A naïve correlation between development and regulation, in this situation, would be positive, suggesting that regulation encourages housing supply. While economists are unlikely to be misled by such a correlation, skeptics in both the broader housing literature and in policy debates will sometimes observe that the most regulated cities also build the most (Baxamusa, 2020; Rodriguez-Pose and Storper, 2020).

This skepticism, moreover, is not easy to defuse with conventional metrics of regulation, because some less regulated cities have little demand, and some more regulated cities have many vacant parcels. The former experience little development despite their leniency (because they lack demand), while the latter see ample development despite their stringency (because they are not yet built out). Controlling specifically for buildout overcomes this problem, and also offers a metric of regulation that lends itself more naturally to policy. There is no obvious path to a city lowering its score on a regulatory index (particularly if the score is a metropolitan area average) but a city *can* expand its zoning envelope.

The paper’s next section sets the stage for our analysis by reviewing previous research on land use regulations and housing markets. Section 3 turns to our data; we describe California’s Housing Element law and emphasize localities’ strategic behavior in complying with it, and also outline our regression approach. Section 4 presents our results, and in the conclusion we discuss policy implications and future research.

## 2. Regulatory barriers: price and supply

The empirical literature on zoning’s role in distorting housing markets is by now substantial.^1^ This literature differs along two important dimensions: the outcome of interest (prices or production) and how regulation is measured. We discuss each in turn.

### 2.1. Prices or production?

Prices and quantity change in the same set of equations, but price is the dependent variable in most land use and housing research.^2^ On the one hand, this disproportionate attention to prices is understandable.^3^ Prices are clearly the relevant outcome for policy. High prices have an intrinsic impact on welfare that low production does not; scholars are more likely to initiate studies because prices are high than because production is low. Production from this perspective is an intermediate outcome, a necessary step toward the outcome of interest. As a result, the idea that regulation suppresses supply is almost always implied, but only sometimes explicitly tested.

Good reasons exist, however, to carry out that explicit test. One involves the endogeneity between prices and regulation. A strong association between regulation and prices might reflect regulation inhibiting production, but also a price premium commanded by regulated environments (Katz and Rosen, 1987; Fischel, 1990). Demonstrating that regulation is in fact associated with less supply would not rule out regulation creating an amenity premium, but would offer some simple support for the mechanism implied in price regressions: regulations raise prices by constraining supply, in addition to mandating higher quality.

Using supply as the dependent variable also creates a more manageable endogeneity problem than does using price. As mentioned above, if regulation can increase prices, but regulated areas also command higher prices (and do so for reasons unrelated to lower production), then tests of regulation’s supply-induced effect on price will have a confirming bias. For any given increase in demand, regulation will make prices rise because it slows building, but also because consumers have a taste for regulated neighborhoods. Left unaddressed, this bias could generate a false positive in a significance test, inflate the coefficients on measures of regulation, or both.

From a policy perspective, this endogeneity may not be a large concern. A regression that incorrectly attributes some or all of regulation’s effect on price to a supply response, when in fact some of that effect is an amenity response, is a mistaken regression. This mistake does not imply, however, that regulation is not increasing prices, or that less regulation will not make housing more affordable. The pathway toward lower prices would be different—the area would become less desirable, holding supply constant, rather than have more supply, holding desirability constant—and this difference could imply different distributional consequences, but fundamentally regulation is still pushing prices up.

From an academic perspective, of course, proper identification is important, and distributional consequences often matter, so researchers do attempt, usually with lags and instrumental variables, to address the endogeneity between regulations and prices (Malpezzi et al., 1998; Mayer and Somerville, 2003; Malpezzi, 2002; Saiz, 2010). Whether these steps adequately control for the price-regulation endogeneity is always a matter of debate (e.g. Davidoff 2016).

A different approach is to avoid price regressions and simply model production. Production, relative to price, is a more straightforward outcome to interpret. This is so because any endogeneity present, given the hypothesized relationship between regulation and supply, will create a *nullifying* bias. Where regulation, for any given increase in demand, will make prices increase by more than they would otherwise, it will make supply rise by less.

This works as follows. We expect more expensive places to produce more housing, ceteris paribus. A simultaneity threat arises because regulation can reduce supply (a negative relationship), but if neighbors become concerned about growth, new supply could also increase regulation (a positive relationship). Left unaddressed, this bias could create a null significance test and/or a positive coefficient on measures of regulation. These results would be unreliable. If, however, the biased regression yielded a negative and statistically significant coefficient on regulation, that coefficient would if anything be too small. Thus in situations where endogeneity is hard to control and identification matters, negative coefficients on supply are more compelling than positive coefficients on price.

### 2.2. Measuring regulation

Housing developments face scores of regulations, not all of them present in all cities, not all enforced with equal intensity in the cities that have them, and not all requiring the same time and effort to satisfy when enforced. Not every city, for instance, demands a traffic analysis for multifamily development. In cities that do require such analyses, moreover, satisfying that requirement in one city might require a single trip to a single department, but in another city might require multiple reviews by multiple departments. For that matter the same regulation within a city might impose much higher costs on some projects than others (e.g., requiring two parking spaces per housing unit matters little for detached single family homes, but can be a binding constraint for small apartments).

Given this variety and complexity, there is virtually no way to fully and consistently capture regulatory stringency in a single measure. Scholars have, however, come up with reasonable proxies. They have examined local zoning codes for the presence of specific measures, or observed changes in cities after new regulations are added (Downs, 2002; Schuetz, 2009; Jackson, 2016). They have also measured the difference between the average and marginal value of land (Glaeser et al., 2005), tracked the frequency of development litigation over time (Ganong and Shoag, 2017), recorded the frequency with which developers request discretionary approvals (Ben-Joseph, 2003), or simply assessed the role of specific bulk regulations like Floor Area Ratio (FAR) (Brueckner et al., 2017; Brueckner and Singh, 2020; Zhang, 2022). While none of these approaches are perfect, they yield broadly consistent results: regulation is significantly associated with less housing production and higher prices.

Nevertheless, a skeptic combing the extant literature could find some reason for doubt. The shadow price approach (Zhang 2022), which tends to focus on specific individual regulations, yields many insignificant coefficients on regulation and some implausible results—for instance, that San Francisco is less stringent than Chicago (Brueckner and Singh, 2020). These null and counterintuitive findings likely arise because emphasizing a single regulation may lead researchers to overlook other avenues cities can use to constrain production.

A broader approach to measuring regulation, and one of the more common ones, is to survey planning staff.^4^ These surveys generally ask about the presence of different regulations, the cost and time to get building permits for different types of development, and rates of enforcement (Glickfeld and Levine, 1992; Ihlanfeldt, 2007; Gyourko et al., 2008; Kok et al., 2014; Pendall et al., 2018; Jackson, 2018; Hilber and Vermeulen, 2016; Gyourko et al., 2021). Researchers use responses to these surveys to build indices of regulation, and then correlate those indices with housing outcomes.

In principle, an index offers a way around the problem of regulatory diversity. Particularly when they are derived with factor analysis, indices can isolate common underlying trends that might plausibly represent regulatory stringency. In practice, the accuracy of the survey responses, and thus the utility of indices built from them, is an open question. One issue is the surveys’ implied faith in planners’ knowledge. Planners preside over the regulatory landscape, so arguably they know that landscape better than others. But superior knowledge is not complete knowledge. As regulations become more complex, it becomes less likely that any one person fully understands them, and perhaps less likely still that this person, if they exist, will be the one to fill out an academic survey.^5^

When researchers directly test for survey errors, the results are not encouraging. Lewis and Marantz (2019) examined eight separate land use regulation surveys in California, and found that the same cities would, in different surveys, report different answers to similar questions. These differences, moreover, could not be explained by time elapsing between surveys. The clearest case comes from 1988: scholars from two separate research projects surveyed the same municipalities. Nine municipalities reported having an urban growth boundary in the first survey but not the second, while five reported an urban growth boundary in the second survey but not the first.^6^

O’Neill et al. (2019), similarly, studied survey responses from eight California cities and found answers that were just wrong. Murray and Schuetz (2019) found the same. To be clear: the direction of error in these cases, when it can be identified, suggests that surveys *underestimate* regulation’s impact. So our point is not that survey errors threaten the literature’s broader conclusions. They do, however, point to the continuing difficulty of accurate measurement.

A final, intriguing twist in the survey literature is that even when planners respond inaccurately to specific objective questions, their broad *impressions* of the regulatory environment seem accurate. Researchers have found, for example, that planners’ responses to subjective questions about development constraints predict housing outcomes better than responses about the presence or absence of specific regulations (Jackson, 2018; Lewis and Marantz, 2019). More specifically, in cities where planners agree that “density restrictions” or “land constraints” are large impediments to new housing, new housing is in fact less likely to be built. This is true even when those same planners do not appear to know what the specific constraints or restrictions in their own cities are.

One interpretation of these results is that planners, even when they don’t know the details of their development codes, do know if their city is a hard or easy place to build. This interpretation is bolstered when we consider that in some ways even the subjective impressions the planners offer are often incorrect. As we discuss more below, no California city actually has a “land constraint” that prevents building. Some cities have little vacant land, but an absence of vacant land is only a hard constraint if redevelopment is physically impossible, which it almost never is. Redevelopment is instead often *legally* difficult; that is the point we turn to next.

## 3. Data and methods

### 3.1. Measurement: process and prohibition

Our empirical approach begins with a basic observation: the extent and pace of developers’ response to rising prices is determined by a market’s price elasticity of supply. Supply elasticities have multiple determinants—including the ease of obtaining raw materials and capacity of the development sector—but cities influence two of them: the complexity of the production process and the availability of a major input (land zoned for residential development).^7^ City policies that complicate the development process (adding hearings or fees), or that reduce the availability of residential land (density restrictions or apartment bans) will make supply less elastic. We can categorize these policies, broadly, as *process* policies and *prohibition* policies. In principle, we can measure them, together or separately, and correlate them with housing outcomes.

In practice, as we mentioned above, such measurement is difficult. A further point is that if we roll process and prohibition policies together into a single metric, as many indices do—perhaps most notably the Wharton Land Use Regulatory Index (WLURI)—we might obscure the mechanisms by which a locality suppresses housing production. This is so for two reasons. First, process policies may matter more when prohibition policies are weaker. In cities where few apartments are allowed, the process for permitting apartments, no matter how cumbersome, is unlikely to be a binding constraint on apartment development. A regression measuring both prohibition and process might show, correctly, that what matters is an overall density restriction, but may also suggest (incorrectly) that a cumbersome process is immaterial. For developers who *do* propose apartments, the process will matter.

Second, process constraints are probably harder to measure than prohibitions. Particularly in places where prohibition does more to limit development (e.g. a typical suburb restricted to single-family homes), process measures may be more prone to reporting errors, because planners will be less familiar with them (e.g. in cities where almost no land is zoned for apartments, planners may not know the steps needed for apartment developers to win approval). Additionally, prohibition measures rarely change. A city’s share of land zoned for single-family housing may change once a decade or less (Gabbe, 2019), and zoning maps that show where apartments aren’t allowed are usually easy to interpret.^8^ Process conditions, in contrast, such as the steps required to obtain a variance, or the circumstances that lead a city to impose cash impact fees or in-kind exactions, change more frequently, and in fact often change from development to development.^9^ Process constraints can change noticeably with each new election to the zoning board or city council, and with changes in executive leadership positions like City Planning Director or City Manager. Folding process and prohibition together may thus combine a set of measurements that are systematically more accurate (though not perfectly so) with a second set that is systematically less so.

In combination, these factors also suggest a third problem: process will be more endogenous to supply than prohibition. Places where developers rarely request approvals are unlikely to adopt complex approval procedures. This will be the case regardless of demand. If demand is high but a city mostly prohibits apartments, and if no one proposes apartments because of that prohibition, then a complicated process for approving apartments is unnecessary. Similarly, if zoning is permissive but demand is low, and no one proposes apartments because of that low demand, a cumbersome process is also superfluous. Places where developers *do* apply to build, conversely, are more likely to respond with process changes than new prohibitions, precisely because process constraints can change more readily. Blanket downzoning is complicated and time-consuming, but cities can quickly respond to unwanted development pressure by enacting more procedures, enacting moratoria, requiring more exactions, or taking longer to review projects (Ionescu, 2022).

The net result is a measurement error that probably biases the size of process coefficients toward zero, and an endogeneity problem that makes their sign more likely to be *positive* (places with more permitting have more onerous processes). The takeaway here is twofold: first, measures of prohibition will suffer from less error than measures of process, although both will be prone to error. Second, because of these issues, and because regulations likely represent an underlying attitude toward new housing, researchers might be able to avoid these problems by finding an alternative approach that measures that attitude more directly. It is this latter point that motivates our use of unbuilt zoned capacity metrics.

### 3.2. Estimates of unbuilt capacity

Our primary measure of regulation is city-reported unbuilt capacity. California law mandates that jurisdictions periodically estimate their unbuilt capacity for new housing. The state requires these estimates as part of the Regional Housing Needs Assessment (RHNA) planning process, which is in turn a part of the state’s Housing Element (HE) law. The RHNA process occurs every eight years; each eight year period is called a “RHNA Cycle.”

Roughly, the RHNA/HE process works as follows. The state assigns each of California’s regional governments a target number of units—a regional “housing need”—that is broken down by household income level.^10^ This target is based on a projection of future growth, and represents the state’s estimate of how much new housing each region will need in the next eight years. The regional governments divide these targets up among their constituent local jurisdictions.

Local governments must then update the HE portion of their general plans to demonstrate that they can “feasibly” add at least as many units as their assigned housing need. “Feasible” has no strict definition, but jurisdictions must identify specific parcels with the potential to hold new housing. Cities complete this exercise, and comply with the law, by presenting an analysis of their unbuilt capacity. The latter must exceed the state-mandated housing target, and it becomes our independent variable of interest.

Compared to typical measures of land use regulation, the HE is useful for three reasons. First, it is compulsory.^11^ Local jurisdictions are legally obligated to complete a housing element, making the nonresponse problem that plagues academic land use surveys largely disappear.

Second, the HE is consequential. The HE is the only part of the general plan subject to state review.^12^ It can affect local growth in the subsequent eight years, and jurisdictions that do not complete HEs face legal consequences. For these reasons, cities give the HE focused attention. Where jurisdictions may devote little time or resources to completing academic surveys, they regularly assign senior planners and/or hire consultants to complete their HEs. The resulting studies are often thorough and lengthy.^13^

Third and most important, the HE is fundamentally a political exercise. The HE is essentially a measure of the city’s broader attitude toward housing. As such, using the HE as a metric can avoid some of the problems that arise from any given regulation being likely endogenous to a city’s larger political environment.

Calling the HE a political exercise is not the same as saying it has no objective data. Cities draw on technical information to justify their Housing Elements, but their estimates of unbuilt capacity reflect strategic behavior at both stages of the process (Monkkonen et al., 2019). In principle, regional governments assign housing allocations to local governments based on need. In practice, local governments lobby regional governments to minimize their allocations—a process enabled in part by local elected officials comprising most regional government leadership. The HE also lends itself to manipulation because allocations of need are based on projections of growth rather than prices, and the projections of future growth are based on past growth (Dillon, 2017). The RHNA thus rewards cities that resist housing; blocking growth in one RHNA cycle yields a lower allocation in the next. The influence of local lobbying was evident in the 5th RNHA cycle, where local governments’ independent estimates of their own growth formed the primary basis of their regional allocation (Monkkonen et al., 2023).

Once assigned a target, cities again have an opportunity to behave strategically, this time when they prepare their HE. The easiest way to demonstrate unbuilt capacity is to identify parcels of vacant land. This is what most low-demand cities do. Almost by definition, however, vacant land is less common in higher-demand cities. A dearth of vacant land does not preclude increasing unbuilt capacity: cities with little vacant land can meet their allocations by rezoning some low-density parcels to allow denser redevelopment. This approach is fiscally almost costless—it can be accomplished with the stroke of a pen^14^—but it is only appealing if cities are open politically to increasing their densities. Until recently they were not: few cities rezoned to meet their housing targets in 2014 (Monkkonen et al., 2023).

Expensive cities that lack vacant land and are *not* open to higher density, and thus want to avoid rezoning, can take a different path: they can identify sites already zoned for multifamily housing that currently hold existing uses, and predict that these sites will be redeveloped into apartments (e.g., rather than allow duplexes in a single-family neighborhood, cities can claim that shopping malls will be demolished and replaced with apartments). These predictions are both common and notoriously inaccurate. A study of sites projected to hold new housing in San Francisco Bay Area HEs found that only 10 % were actually developed over the planning period, and that a majority of the development that did occur in these cities took place on sites not even listed in the HEs (Kapur et al., 2021).

This inaccuracy could arise from error, from strategic behavior, or both. Anecdotally, strategic behavior appears the more likely culprit—especially but not exclusively in earlier (pre-2022) cycles (Monkkonen et al., 2023).^15^ Early in the 6th cycle, for example, the City of Vista listed both its own City Hall and public library as sites likely to be torn down and redeveloped as high-density housing. South Pasadena, another affluent suburb dominated by single family homes, predicted that every major grocery store within its borders would be redeveloped into affordable housing. And the pastor of a church in the wealthy enclave of La Canada Flintridge told a local newspaper that she agreed to let the city list church property as a site for future affordable housing, since “everyone knows” it will “never be built.” (An exact quote: “We thought … ‘what’s the harm in letting our property be listed as one of the imaginary sites where it could be built, for the sake of submission to the state’? So we did the city a favor.”) (Pringle, 2022).

From our perspective, this inaccuracy, and the fact that it arises from political gamesmanship, is advantageous. Were the poor predictions simply random error, they would tell us little about housing policy. Because they arise from strategic behavior, however, they offer a window into antipathy to housing. Put another way: one reason the predictions are inaccurate is that many local governments do not want to allow more housing; they want to meet their planning targets with minimal risk of actual redevelopment. Anecdotally, these cities often report only their legally required level of unbuilt capacity: in each cycle they have just enough room to hit their assigned housing targets, but no more. They are, in their own judgment, “built out.”^16^

It is worth reiterating that buildout is a subjective judgment. No California cities are in fact physically built out. Even in places with topographical constraints, at a minimum almost every single-family home can be replaced with a duplex, and many can be subdivided into apartments. The modern elevator was invented in 1903, and engineers have known for half a century how to construct buildings over 100 stories tall. Most of urban California is between one and two stories, suggesting ample room for vertical expansion. Indeed, a 2016 study identified viable space for more than five million units in California’s existing urban neighborhoods (Woetzel et al., 2016). Buildout is a political construct, and cities that determine themselves closer to buildout are presumably more hostile to development.^17^

Understanding this point can help resolve some otherwise unusual findings in the literature. Jackson (2018), for instance, argues that “Housing supply in California cities is made inelastic by land constraints, not regulation.” But most land constraints *are* regulations. Jackson draws his conclusion from responses to a survey question about “developable land.” With few exceptions, however (such as steep hills and seismic areas), “developable” is a subjective determination, and assertions that a parcel is undevelopable often rest on an assumption that once a parcel has a structure on it, it cannot be redeveloped at higher density. Virtually the entire history of urbanization, of course, suggests that this assumption is inaccurate—Manhattan once had large parcels dotted with farmhouses. These farmhouses were first subdivided into apartments, and then demolished and replaced with higher density buildings. Planners responding to surveys might *believe* buildout represents a land constraint, but it is more accurately thought of as a political constraint placed on land.^18^

We obtained the estimates of unbuilt capacity for 414 out of 482 of California’s cities for the period 2014–2021, and estimates specific to unbuilt multifamily capacity for 346 cities. (Some cities did not report their multifamily capacity independently.)

### 3.3. Regulatory process and prohibition indices

We supplement our unbuilt capacity measure with three conventional metrics of regulation, one measuring process, and measuring prohibition, and one—the Wharton Index—that combines the two. We discuss these in turn.

We build our Process Index from eight questions in Jackson’s (2018) survey of 450 California municipalities. These questions ask about the number of regulatory bodies that must grant permission for a residential development to proceed, whether planning staff can grant some development approvals or if elected officials or commissions must weigh in, how often permit-granting entities meet, and so on. We transformed responses to each question into binary variables, to reflect above or below average burden, and then sum the variables to obtain our index. The index ranges from one (low process burden) to eight (high) and the median city scores four. Appendix B contains more detail.

Our Prohibition Index comes from the Mercatus-Augmented Terner California Housing Regulation (MATCHR) survey, which covers over 250 California jurisdictions (Furth and Gonzalez, 2019). MATCHR is, as its name suggests, the Mercatus Institute’s expanded version of the land use survey carried out by UC Berkeley’s Terner Center. MATCHR’s creators converted the survey results into an index using a factor analysis that condenses responses about zoning rules into a single number.

This approach to building MATCHR is essentially the same as the approach used to construct the better-known WRLURI (Gyourko et al., 2008; Gyourko et al., 2021). For our purposes, however, WRLURI and MATCHR differ along two important dimensions. First, MATCHR is more narrowly focused than the WRLURI. Where WRLURI includes 12 subindexes in three categories, the MATCHR prohibition index uses only five inputs: single-family minimum lot sizes, single-family parking requirements, an index of single-family setbacks, the share of residential land zoned for multi-family housing, and an index of sixteen multifamily regulations (including parking requirements, setbacks, open space rules, and other development standards). The MATCHR factor analysis identifies a single latent (unobserved) factor underlying these five inputs (which represent more than five regulations, since two of the inputs are themselves indexes). For the roughly 250 cities with data, the MATCHR index ranges from −1.5 to 3.9 with a median of −0.14.

Second, the WRLURI includes not only process and prohibition variables, it also includes, in some of its subindices, a series of metrics that are essentially outcomes—such as the number of rezoning permit applications that occurred in a city.

We expect measures of regulatory prohibitions to correlate negatively with unbuilt capacity, since places with strict density restrictions will, ceteris paribus, have less legal room for new housing. This expectation turns out to be correct (in Appendix C, Table C1, we present these and other correlations between measures of regulation). We also expect, however, that unbuilt capacity will capture more information about development potential than does an index of regulatory prohibitions, because unbuilt capacity accounts for existing buildings. For example, a city zoned entirely for single family homes could have plentiful undeveloped land. An index of prohibitions might record this city as stringently regulated, but it could still easily add housing.

The MATCHR, like the WRLURI, illustrates both the advantages and disadvantages of regulatory indices. Both surveys condense a tremendous amount of information into a single number, which is a benefit. They also, however, replace the black box of regulation with the black box of an index. MATCHR, moreover, appears to be sensitive to outliers. Furth and Gonzalez (2019) report that MATCHR’s correlation with housing development appears to be driven primarily by “extreme values”—a handful of affluent suburbs with very low-density zoning. MATCHR’s sensitivity to outliers may in part explain why it is only weakly correlated (0.10) with the 2018 values of WRLURI for the 98 municipalities observed in both surveys. Another explanation for this low correlation, of course, is that the WRULI includes measures of both process and prohibition, while MATCHR is just a prohibition index.

### 3.4. Models

We assume, and theory suggests, that more housing construction will occur in cities with higher rents (with rents being a proxy for returns on development), unless regulation in some form prevents it. Our regressions thus examine associations between permitting, regulation, and demand. Our models do not control for endogeneity, but our decision to model supply should mean that any endogeneity will favor smaller coefficients or null significance tests.

Our first regressions test different variations of the hypothesis that cities with more onerous regulations will permit less new housing. The variations alternate the measures of regulation: unbuilt capacity, a prohibition index, a process index, and the composite WRLURI. Our regressions separately examine all building permits issued in a city between 2014 and 2019, and multifamily permits. The models take the following form:

$$Ln(\text{Permits}\;2014-2019)=\alpha +\beta_{1}ln(\text{Rent})+\beta_{2}Reg+\beta_{3}\text{City}+\beta_{4}\text{Dem}+\text{Metro}+e$$

Where permits are either all permits or multifamily permits, rents are measured in 2013, *Reg* is one of the regulatory indexes (unbuilt capacity, prohibitions, process, or the composite WRLURI), *City* denotes a vector of city controls including city size, job accessibility, population density, demographics, and recent change in rents, and *Metro* is a metropolitan area fixed effect. As discussed above, we expect coefficients on the Process Index to be biased toward zero. We also recognize that the relationship between supply and regulation likely has nonlinearities that won’t be fully captured with our functional form (though most of the literature on this topic shares this limitation).

Multifamily housing production was roughly half of total housing production in California during our study period, but nearly one third of the cities in our sample built no multifamily units during that time.^19^ Because so many cities did not permit any multifamily units, for the multifamily permitting regressions we use a Heckman selection model, identified on functional form. We could not identify any measurable characteristics of a municipality associated with *any* multifamily permitting that would not also be correlated with the *amount* of permitting.

The second set of regressions interact our measures of regulation with rent. These terms explicitly test the idea that regulation binds in the presence of demand. That idea is far from controversial, but we hypothesize that it will be harder to demonstrate with a conventional regulation index. Some cities that are permissive on paper may have a limited number of undeveloped parcels, and thus fewer places to put buildings that they nominally allow. Similarly, two cities that allow multifamily development on half their land might look almost identical in a Prohibition Index. But if one has an abundance of vacant land and the other has none, the latter is excessively regulated and the former may not be. A regression where Prohibition is the coefficient of interest would not capture this nuance, but a regression examining unbuilt capacity could.

We report descriptive statistics for the variables we will use in our analysis in Table 1, and data availability by region for the different regulatory variables in Table C2.

## 4. Results

### 4.1. Understanding unbuilt capacity

Before modeling permitting, we examine our main independent variable of interest, unbuilt capacity, by describing it and considering its determinants. A city’s total unbuilt capacity that city’s is its estimate, reported in its Housing Element, of the number of new housing units its existing zoning allows. For the median city, total unbuilt capacity in 2014 was 1641 housing units. As a share of existing housing units, the median jurisdiction reports sufficient unbuilt capacity to grow its housing stock by 13 %. Considerable variance exists around this median. The city with the lowest unbuilt capacity reports being able to add only 1 % of its current stock, while the city with the highest unbuilt capacity can grow its stock over 200 %. Table C3 reports the distribution of this variable and our other main variables of interest, as well as housing growth targets, which for the median city represent 6 % of housing stock.

**Fig. 1.**
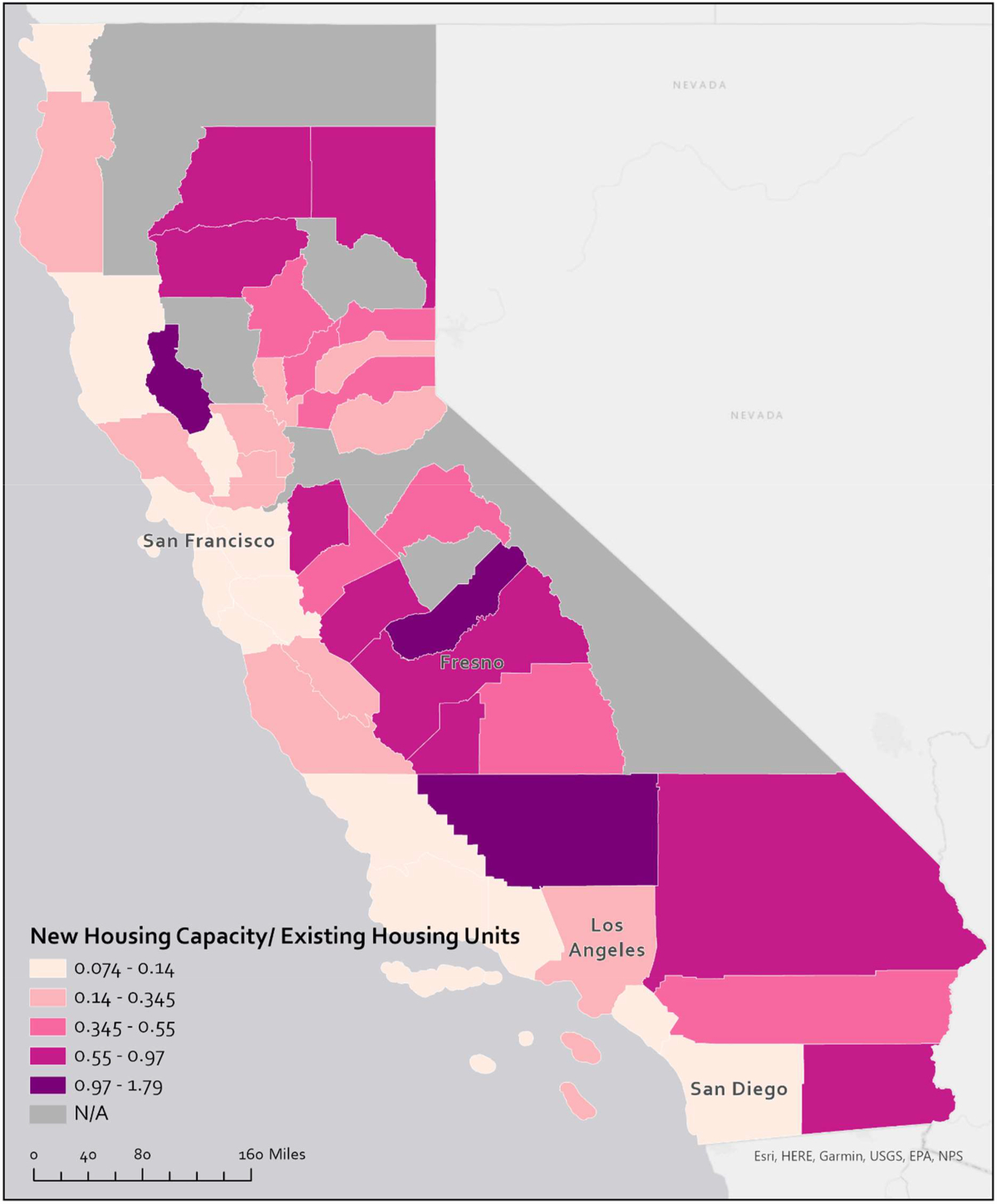
Median values of unbuilt capacity as a share of housing units by county.

**Fig. 2.**
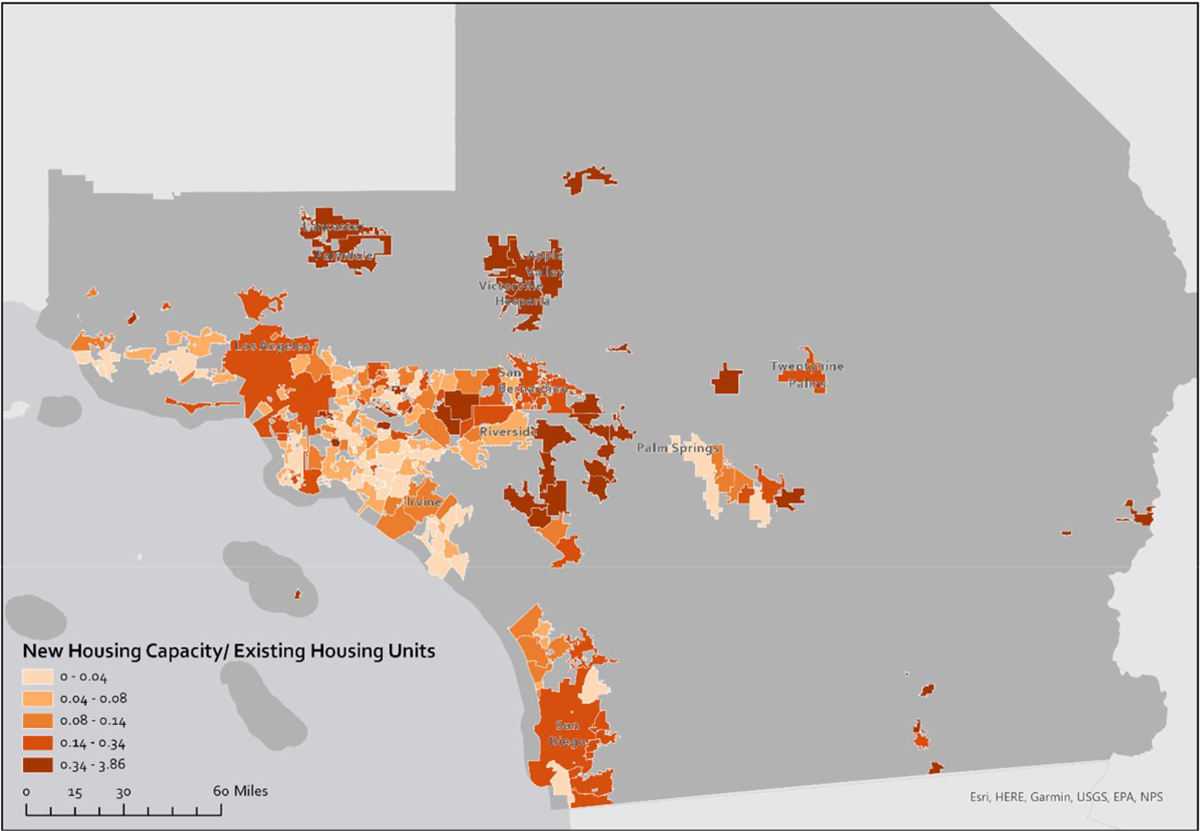
Unbuilt capacity as a share of existing housing units in Southern California.

Fig. 1 is a choropleth map of California counties that illustrates the capacity for new housing as a share of existing housing. The map clearly shows that cities in coastal counties, where the demand for housing is higher, report substantially less capacity (on average) for new housing development. Fig. 2 shows unbuilt capacity as a share of existing units for cities in Southern California. The same pattern we see across regions in Fig. 1 exist within the region in Fig. 2. Coastal and expensive jurisdictions report less space for growth in their zoning codes, with the exception of large cities like Los Angeles and San Diego. However, the Housing Element of the City of Los Angeles suggests that within larger cities there is a similar imbalance of unbuilt capacity and demand (City of Los Angeles, 2021).

**Fig. C1.**
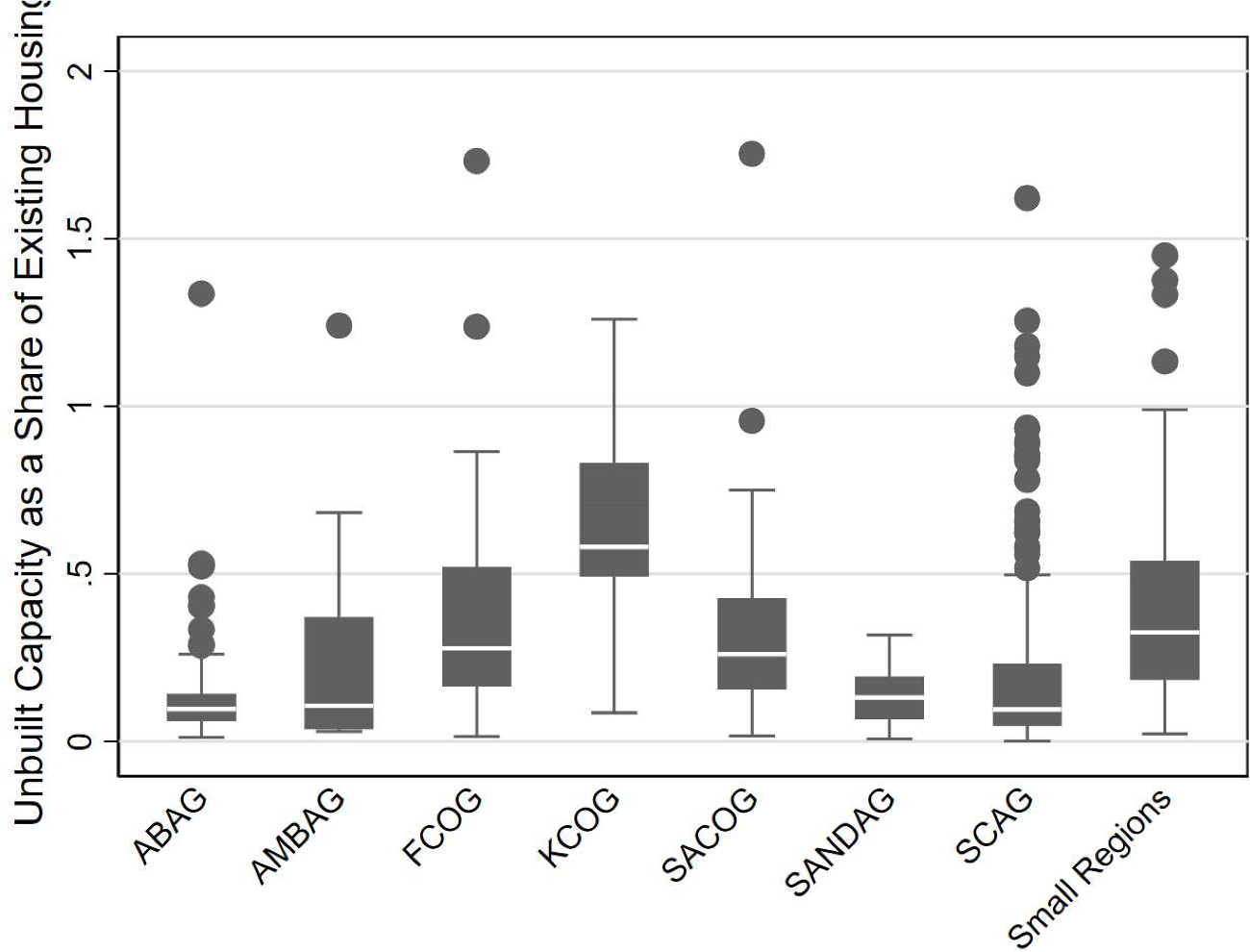
The distribution of unbuilt capacity as a share of existing housing across regions. Note: Regions are Association of Bay Area Governments (ABAG), Association of Monterrey Bay Area Governments (AMBAG), Fresno Council of Governments (FCOG), Kern Council of Governments (KCOG), Sacramento Area Council of Governments (SACOG), San Diego Association of Governments (SANDAG), and Southern California Association of Governments (SCAG).

Comparing Figs. 1 and 2 also suggests that unbuilt capacity varies less across regions than within them. A coefficient of variation confirms this: the coefficient for unbuilt capacity as a share of existing housing across regions is 0.73, whereas within regions it averages 1.16. Only in the San Diego region is there less variation across municipalities (at 0.63) than across the state’s regions.^20^ In Fig. C1, we present a graph of the distribution of unbuilt capacity as a share of existing housing across regions.

The varied distribution of unbuilt capacity is consistent with the idea that these numbers arise from a combination of history and politics. Because the RHNA process only asks that cities find *room* for new housing (it does not demand physical units) in the absence strategic behavior the variation across cities in estimated capacity should be low (cities can always rezone). The *composition* of the capacity might be heterogenous, in that some cities will point to vacant land and others will point to room in the zoning envelope, but capacity itself should not vary. That is not what we observe.

Table 2 tests the idea that cities with more demand and more existing density report less capacity, presenting results from regressions of the log of unbuilt capacity on potential determinants.^21^ These are existing density, housing values, an index of regulation, the age of housing stock, and demographic characteristics often associated with opposition to new housing: share of residents over 65 years old, share homeowners, and share White (Fischel, 2001; Einstein et al., 2020; Fang et al., 2022).

The results show that denser cities have less unbuilt capacity, as do cities with more valuable housing, a larger share of housing built before 1990, and more homeowners. Neither the share of residents over the age of 65 nor the share White are associated with unbuilt capacity, suggesting that if regulatory buildout reflects NIMBYism it is more closely aligned with home values and ownership, than with demographic attributes that tend to be correlated with conservative preferences (Fischel, 2001; Kahn, 2011; Manville 2021).

None of the regulatory indexes have statistically significant associations with unbuilt capacity. This is so in our previously reported bivariate correlations, and we see the same here in our regressions with controls. We interpret these results as illustrating the importance of measuring buildout. As we suggested above, cities can score low on some measures of regulation because their zoning allows a substantial amount of multifamily housing. If all or most of their multifamily zoned parcels are built out, however, the fact that multifamily housing is allowed will not be a strong determinant of whether the city adds units. What will matter is the potential to redevelop, at higher density, sites that are already built on. In these situations, estimates of buildout will be more telling. Such situations do arise, moreover, in our data. There are cities in California with strict regulations but many vacant parcels, as well as cities with permissive regulatory environments but parcels built out to their zoned maxima.^22^

The distinction between prohibition and buildout explains Murray and Schuetz’s (2019) finding that cities in California with high rents did not build more apartments than other cities. These cities do not prohibit rental units (as evidenced by their rental stock) but they have either control for the log of population. affirmatively downzoned, or failed to upzone, and thus given themselves little room to permit building types they technically allow.^23^

### 4.2. Analyzing permitting

We now turn to new housing supply. Table 3 reports the results of five models, where the dependent variable is the log of all permits issued, regressed on our measures of regulation with controls for population, population density, rents in the year preceding the permit data, jobs accessibility, share multifamily in the city, race/ethnicity, and the recent (2009–2013) change in rents. We report the full model results in Table C4.

Cities with less unbuilt capacity permitted less housing. A 10 % decrease in unbuilt capacity is associated with nearly 4 % fewer housing permits (Model 1). The coefficient on stringent prohibitions is negative, but not statistically significant (Model 2).^24^ The Process Index coefficient is neither statistically significant nor large (Model 3). Its near-zero magnitude suggests that an onerous permitting process has little economic significance with respect to housing production. As discussed previously, this result may reflect Process’s greater endogeneity to supply.

Model 4 combines these three measures of regulation. Doing so reduces the sample size dramatically (to 135 observations), mostly as a result of missing values for the Process Index. Unbuilt capacity has a similar coefficient but the coefficient on the Prohibition Index grows substantially. This change in coefficient, however, appears primarily to be a result of the constrained sample rather than the interaction of the indexes—if we estimate Model 2 on this much smaller sample, the Prohibition coefficient is similarly large. Nonetheless, the fact that both measures of regulation are significant reflects the fact that specific regulatory prohibitions still matter beyond the more comprehensive reflection of political attitudes towards new housing development and buildout captured by unbuilt capacity.

In Model 5, we examine the correlation between permitting and the WRLURI index. The coefficient is positive but not statistically significant, most likely because (as we noted above) the Wharton Index contains more information on process than prohibition.

Table 4 repeats the analysis of Table 3, but uses the log of multifamily permits from 2014 to 2019 as the dependent variable. We report the full results in Table C5 and the results of the first step in the Heckman selection model in Table C6. Fewer cities reported their multifamily capacity and many cities permitted zero multifamily units, so the sample size is smaller.

The unbuilt capacity coefficient shrinks slightly in the model of multifamily permitting, though it remains substantial. A 100 % decrease in a city’s unbuilt capacity is associated with 24 % fewer multifamily permits issued (Model 1). The coefficient on the Prohibition Index, on the other hand, grows compared to its size in the models predicting all permits, and is statistically significant (Model 2). A one-unit increase in the Prohibition Index (close to its standard deviation of 0.87) is associated with 35 % fewer permits. Given that several components of the Prohibition Index directly measure restrictions on multifamily housing, this result is expected. The coefficient on the Process Index is again positive and in this case significantly associated with multifamily permitting (Model 3). As discussed previously, this is likely due to endogeneity: more permitting can lead cities to adopt more onerous processes.

As with the previous models, we run a model that includes the Process, Prohibition and unbuilt capacity measures together (Model 4). This again reduces the sample size substantially, in this case to only 92 observations. In this model, the coefficient on unbuilt capacity is smaller by about half, and the coefficients on the other indexes increase substantially. As with Model 4, the changes to the coefficients result more from the smaller sample rather than an interaction between the terms. Running Models 1–3 on this small sample yields similar coefficients and significance. Again, when we estimate a model using the WRLURI, it is negative but not statistically significant (Model 5).

There is no single correct way to control for city size. In the above models, we control for city size on the right hand side. Given the slight tendency for larger cities to have higher rents and more multifamily housing, we also run models that control for city size on the left hand side, using the permitting rate—the number of permits issued from 2014 to 2019 per 100 housing units in 2013—as the dependent variable. Tables C7 and C8 report the results of these regressions of permitting rate on the same set of independent variables as Tables 3 and 4. The unbuilt capacity measure is consistently significant and large in these models. The index of prohibitions attains significance in the model of all permits but loses significance in the model of multifamily permits. Coefficients for the process index and WRLURI are similar to models in Tables 3 and 4.

As an additional robustness check, we estimate models that use housing values to capture demand instead of rents. The results, reported in Table C9, are similar in both the significance and direction of coefficients as the models using rents to measure demand.

### 4.3. Interacting rents with regulation and unbuilt capacity

In Table 5, we present regressions of log permits on controls as well as the interaction between regulation and rents. Here we see our most substantial finding: unbuilt capacity matters more in expensive cities, and rents are more likely to predict new supply in cities with space for new housing in their zoning code. Cities that have ample space for new housing in their zoning code but also have low rents do not permit much housing. It is only in cities that have both high rents and unbuilt capacity that we see substantial permitting. This result is intuitive: developers want to build where returns are highest, so cities with low rents permit very little housing regardless of their regulatory landscape.

The interaction term is not significant for the survey-based measures of regulation, suggesting that these measures correlate with production in the same way in high and low-rent cities.^25^ This finding, in turn, suggests a deficiency in the prohibition measure: it implies that land use regulations will bind equally regardless of demand. This deficiency may owe to the issue we mentioned earlier: some tightly zoned cities have many vacant parcels, while some cities with many parcels zoned for multifamily may not.

**Fig. 3.**
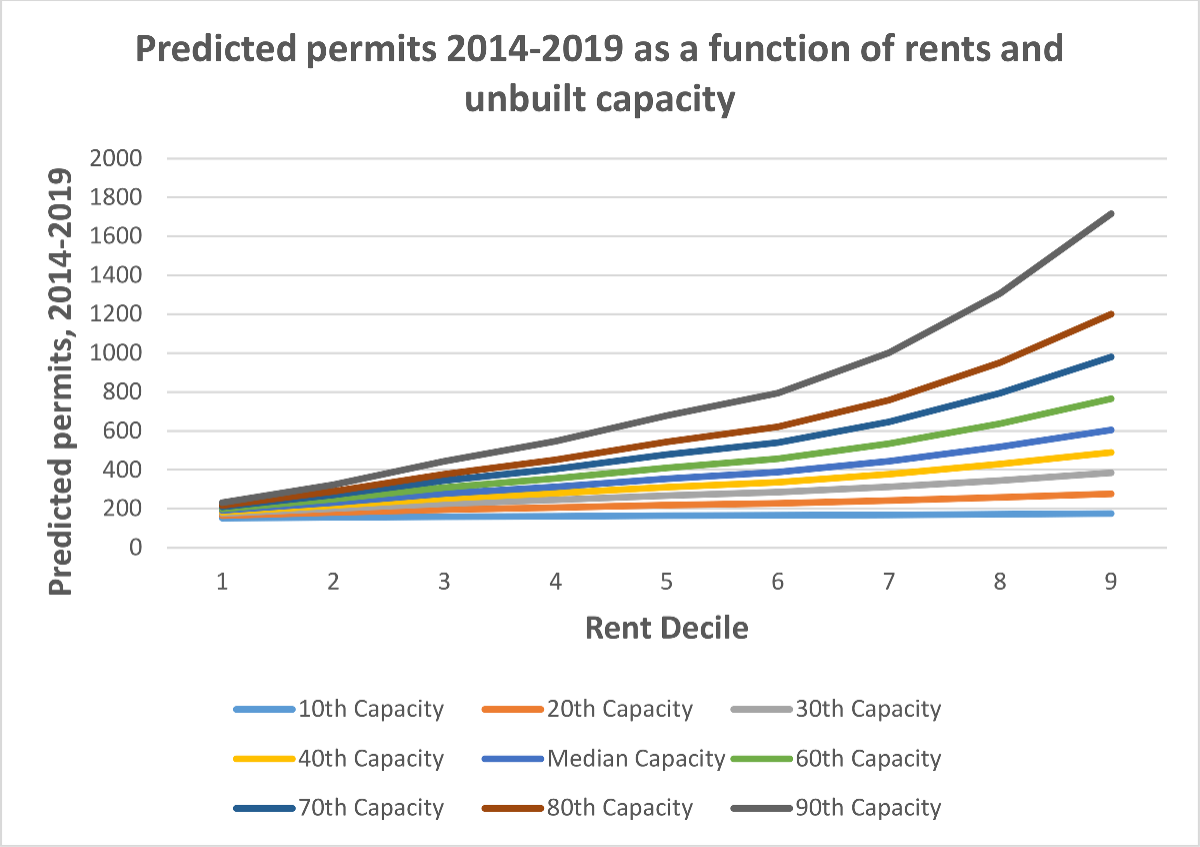
Predicted total permits as a function of rent and unbuilt capacity.

**Fig. 4.**
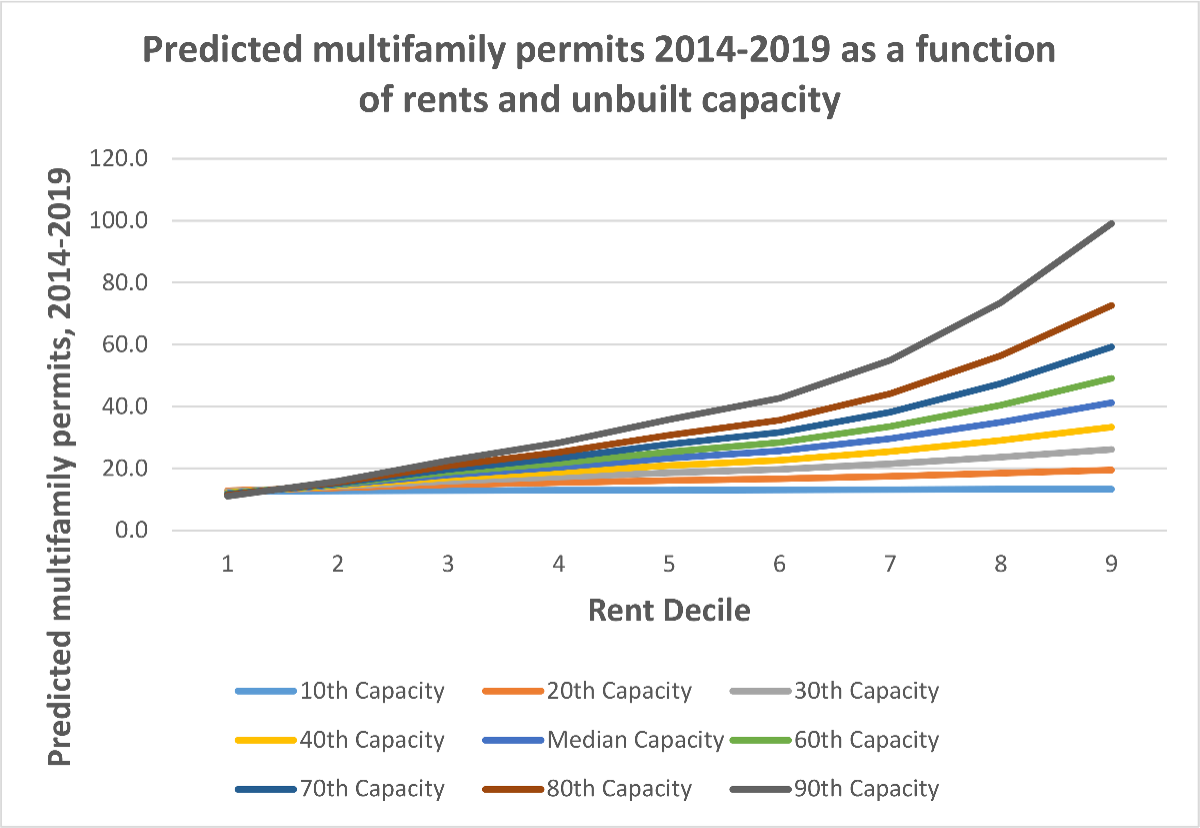
Predicted multifamily permits as a function of rent and unbuilt capacity.

We illustrate the interactions between capacity and rents in Figs. 3 and 4, which show the models’ predicted permitting levels (of all housing and of multifamily housing) as a function of both rents *and* unbuilt capacity. The positive interaction term means that unbuilt capacity matters more at higher rent levels; it suggests, in short, that housing will only be permitted in large numbers by cities where rents are high *and* the zoning code has space. We observe an inflection point for cities above the 60th percentile in rent, which in 2013 was roughly $1520. Above this rent level, the relationship between unbuilt capacity and permitting increases substantially.

As an example, consider two cities, both of which have rents at the 30th percentile (roughly $1100) but one has unbuilt capacity at the 20th percentile and the other at the 80th. The city with more space is predicted to permit nearly twice as many housing units between 2014 and 2019, 378 units compared to 196. This difference is substantial. But now consider two cities at the 80th percentile of rents (roughly $1900), with one city having unbuilt capacity at the 20th percentile and the other at the 80th. Here the model predicts that the city with more space will permit *four times* as many units, 960 units compared to 240.

**Fig. C3.**
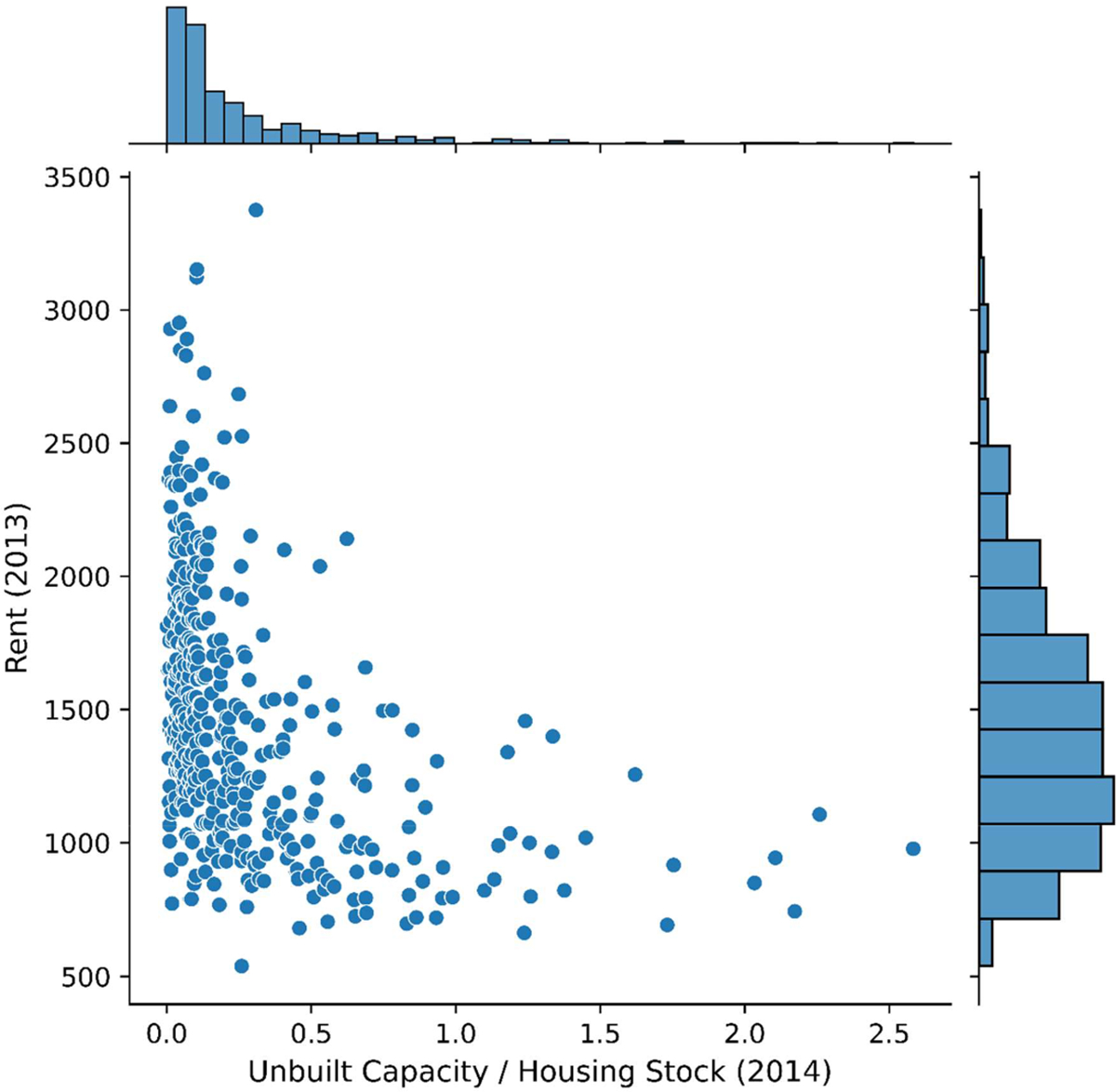
Joint frequency distribution of rent in 2013 and unbuilt capacity as a share of existing housing.

Relatively few places in our data, however, are above this rental inflection point. Of the over 100 cities in the top quartile of rent, only 13 are also in the top quartile of unbuilt capacity and only five are in the top quartile of capacity as a share of existing stock.^26^ (This may help explain why, of the 40 cities with the highest rents, 29 were in the bottom quartile for housing growth). Only one city, Irvine, is in the top 10 % of both rents and capacity. We present the joint frequency distribution of rents and unbuilt capacity in Fig. C3.

Fig. 4 shows that the interaction between rent and capacity in predictions of permitting is similarly important for multifamily housing—a result that owes largely to the limited association between unbuilt capacity and permitting in cities with low rents. The model predicts that cities in the 10th percentile of rents with less unbuilt capacity will actually have higher levels of permitting. At the 30th percentile of rents, however, moving from the 20th to 80th percentile of unbuilt multifamily capacity is associated with a 40 % increase in permitting, whereas at the 80th percentile of rents the model predicts a 200 % higher level of permitting when moving from 20th to 80th percentile capacity.

**Fig. 5.**
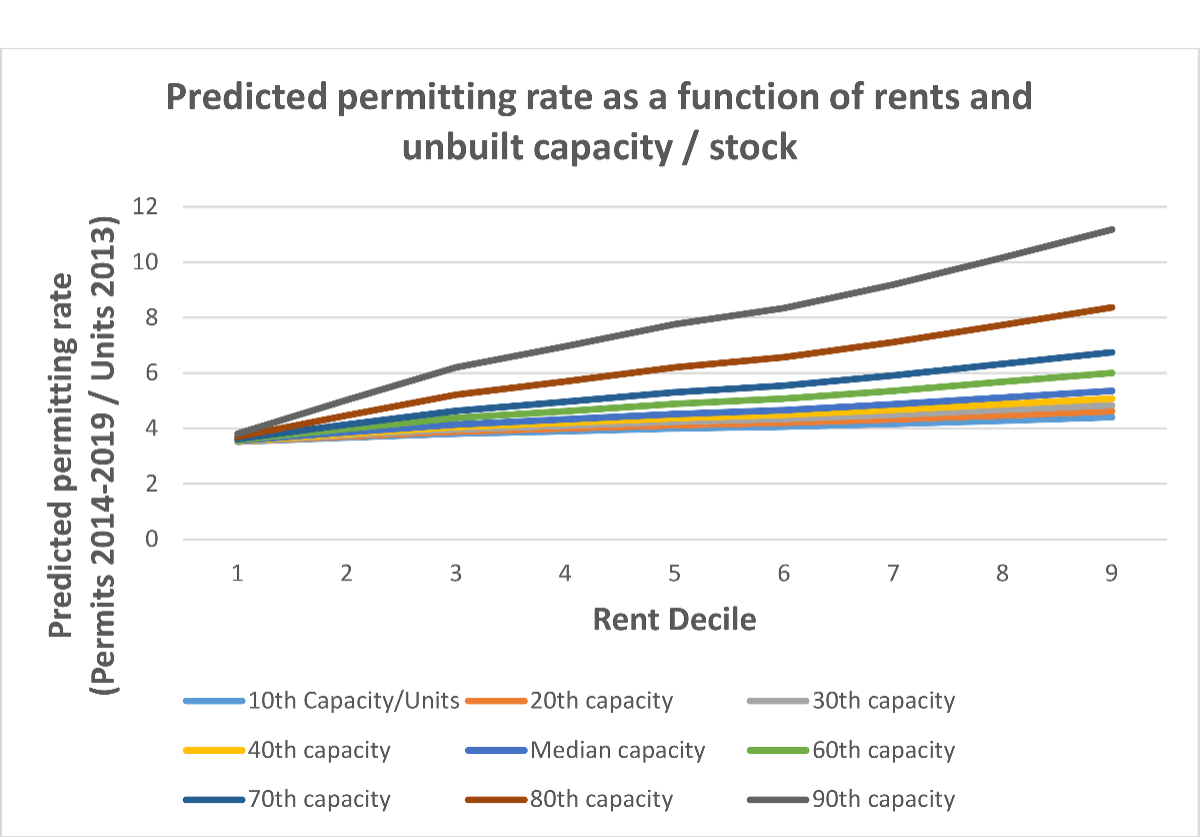
Predicted permitting rate 2014–2019 as a function of rent and unbuilt capacity / stock.

**Fig. 6.**
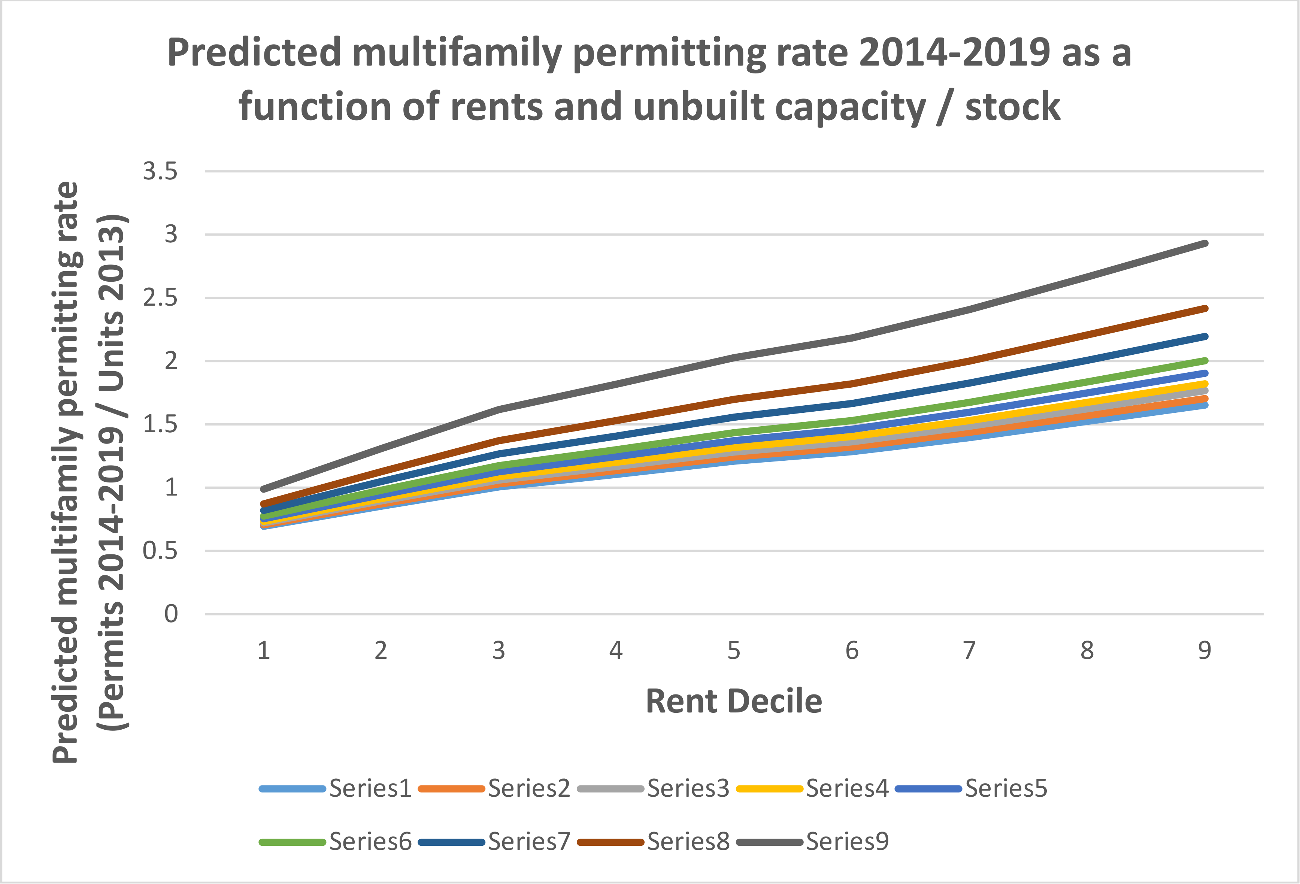
Predicted multifamily permits as a function of rent and unbuilt capacity.

A natural concern about our results is that a small handful of outlier localities (bigger cities with higher rents) might be driving our finding. To address this concern, Figs. 5 and 6 replicate Figs. 3 and 4 but use predictions of the permitting rate, or the number of permits issued between 2014 and 2019 as a share of the 2013 housing stock. These figures suggest outliers are not driving our results. Permitting rates show a less dramatic, but nonetheless substantial relative increase in permitting for high rent places as capacity increases.

Using the same examples as above (cities at the 30th and 80th percentile in rents moving from the 20th to 80th percentile capacity), we see a 70 % increase in permitting rate for higher rent cities that increase capacity but only a 30 % increase for lower rent cities.

## 5. Discussion and conclusions

There is strong reason to believe that land use regulations increase housing prices, but empirically testing that relationship is difficult. Regulation is notoriously difficult to measure: different cities have different rules, not every regulation is equally costly in every context, and regulations that are individually benign can, in combination, become burdensome. Even assuming regulation can be measured, moreover, endogeneity can confound attempts to persuasively link it with price. Regulated places might, for reasons unrelated to supply, command a price premium.

We address these obstacles in two ways. First, we model regulation’s impact on supply, rather than price. Supply is also endogenous to regulation, but its endogeneity creates a nullifying rather than a confirming bias, meaning that our results may, if anything, be conservative estimates of regulation’s effect.

Second—and this is our primary contribution—we eschew conventional measures of land use regulation and instead measure the underlying political sentiment that regulation represents, which is a locality’s willingness to accept new housing. A proxy for that sentiment is available to us because California state law requires cities to periodically estimate how much additional housing they can hold. The judgment that informs those estimates is largely political. Even cities with steep terrain and little vacant land can substantially increase their unbuilt capacity by allowing parcels zoned to hold one unit to instead hold two. Given that many local governments zone mostly for single-family homes, such a change could almost double allowed housing capacity, and even do so without meaningfully changing building footprints. Differences in unbuilt capacity represent differences in openness to development, and these attitudes manifest in (hard-to-measure) regulations.

Using simple models, we show that this measure has associations with new housing supply that are equal to or larger than the associations between supply and conventional regulatory indices. We also show that this buildout measure captures regulation’s interaction with demand in ways that conventional indices do not.

One could draw the wrong lesson from these results, and arrive at the zoning version of “guns don’t kill people, people kill people.” Such a conclusion, that regulations are irrelevant, would be mistaken. The fact that underlying causes matter doesn’t mean proximate causes don’t. Some regulations really are more burdensome than others. Our point is only that a regulatory regime is the legal embodiment of a political atmosphere, and metrics that better reflect that reality may also better predict housing outcomes.

Such metrics may also lend themselves more readily to policy. The literature on regulation and housing supply is not new, but a political movement determined to apply its lessons is (e.g. Dougherty, 2020). The rise of pro-housing activists determined to act on economic research casts into sharp relief one limitation of regulatory indices: it is difficult for local or state officials to understand and change them. A zoning envelope, as a simple measure of how much housing a city allows, is different. It is imperfect, but almost certainly more tractable. Although the measure we use in this paper is limited to California, researchers could estimate unbuilt capacity based on land use regulations and existing buildings in other places.

Our results suggest, in fact, that state governments interested in more housing production would do well to focus on increasing the zoned capacity in expensive cities (and the expensive neighborhoods of these cities). States could directly incorporate this lesson, for example, into the allocation methodologies of fair share housing plans, by allocating higher shares to places with higher rents. Alternatively, if state governments considered targeted preemption of local zoning based on proximity to public transportation, they should do so guided by not just proximity to transit but also demand. State preemption of exclusionary zoning will have a larger impact in high rent cities and neighborhoods. (Our analysis does not tell us directly that the relationships we observe apply to high rent neighborhoods in relatively inexpensive cities, but we anticipate that they do.)

For the same reason, our results also speak to concerns that zoning reform will harm lower-income communities, by subjecting them to waves of development and gentrification. This concern often leads to debates over whether new development does in fact harm lower-income neighborhoods, and whether it accelerates or delays gentrification. Our analysis, however, suggests that widespread upzoning would concentrate new development in places where rents (and thus presumably incomes) are already high.

## Figures and Tables

**Table 1:** Descriptive statistics.

Variable	Median	Standard deviation

Unbuilt capacity (units)	1641.00	18,345.50
Multifamily unbuilt capacity (units)	956.00	7962.00
Unbuilt capacity (units) / Housing units	0.13	0.56
Prohibition index (MATCHR)	−0.14	0.87
Process index	4.00	1.76
All permits 2014–2019	271.00	4712.71
All permits 2014–2019 / 100 housing units (2013)	2.59	5.22
Multifamily permits 2014–2019	42.00	3929.70
Multifamily permits 2014–2019 / 100 housing units (2013)	0.50	2.40
Median rent, 2013	1374.50	500.04
Change in rents, 2009–2013 (%)	0.21	0.15
Median housing value, 2013	403,100.00	361,532.09
Housing built before 1940 (%)	0.05	0.09
Housing built before 1990 (%)	0.61	0.20
Population density (per square mile)	3607.99	3453.60
Multifamily housing (%)	0.36	0.16
Total population	31,864.50	211,072.92
Job accessibility (within 20 min)	14,910.33	28,576.71
Homeowners (%)	0.53	0.14
Over 65 years old (%)	0.13	0.07
Hispanic population (%)	0.32	0.26
Asian population (%)	0.18	0.14
Black population (%)	0.02	0.05
White population (%)	0.46	0.25

Source: California Housing Elements, Furth and Gonzalez (2019); Jackson (2016); US Census.

**Table 2:** OLS results; dependent variable is the log of unbuilt capacity (in units) in 2014.

Variable	1	2	3	4	5

Population density (log)	−0.568***	−0.462***	−0.392**	−0.417***	−0.364
	(0.086)	(0.089)	(0.161)	(0.141)	(0.228)
Median housing value (log)	−0.494***	−0.415***	−0.614***	−0.369**	−1.058***
	(0.172)	(0.156)	(0.211)	(0.166)	(0.332)
Job accessibility	−0.104	−0.029	0.119	0.057	0.144
	(0.067)	(0.062)	(0.094)	(0.137)	(0.162)
Housing built before 1940 (%)	0.180				
	(0.643)				
Housing built before 1990 (%)		−1.717***	−1.568**	−2.302***	−1.749*
		(0.446)	(0.708)	(0.543)	(0.992)
Prohibition index			−0.116		
			(0.093)		
Process index				−0.0275	
				(0.038)	
WRLURI					0.124
					(0.111)
Homeowners (%)	−0.979**	−1.453***			
	(0.446)	(0.426)			
Over 65 years old (%)	−1.336	−0.628			
	(0.967)	(0.921)			
White population (%)	−0.0794	0.00579			
	(0.348)	(0.332)			
Constant	9.767***	9.108***	9.350***	7.462***	15.16***
	(1.974)	(1.816)	(2.572)	(2.233)	(4.528)
Observations	413	413	228	230	147
*R*-squared	0.65	0.67	0.64	0.64	0.61

Notes: ***, ** and * indicate significance at the 0.01, 0.05, and 0.10 levels. Robust standard errors in parentheses. Models include metropolitan area fixed effects and a control for the log of population.

**Table 3:** OLS results; dependent variable is the log of all permits 2014–2019.

Variables	1	2	3	4	5

Unbuilt capacity (log)	0.362***			0.333***	
	(0.075)			(0.119)	
Prohibition index		−0.101		−0.371**	
		(0.132)		(0.184)	
Process index			0.040	0.007	
			(0.048)	(0.069)	
WRLURI					0.074
					(0.135)
Median rent, 2013 (log)	0.906**	0.355	−0.430	0.642	−0.722
	(0.409)	(0.613)	(0.607)	(0.792)	(0.916)
Population (log)	0.905***	1.259***	1.277***	0.961***	1.260***
	(0.101)	(0.077)	(0.099)	(0.178)	(0.136)
Population density (log)	−0.157	−0.530***	−0.113	−0.473**	−0.027
	(0.134)	(0.160)	(0.183)	(0.221)	(0.267)
Job accessibility (log)	−0.072	0.063	−0.412***	−0.239	−0.227
	(0.091)	(0.126)	(0.153)	(0.171)	(0.217)
Constant	−11.180***	−6.392	−0.270	−5.887	0.755
	(2.989)	(4.277)	(4.109)	(5.595)	(6.440)
Observations	404	225	229	133	147
R2	0.669	0.646	0.565	0.639	0.593

Notes: ***, ** and * indicate significance at the 0.01, 0.05, and 0.10 levels. Robust standard errors in parentheses. Models include metropolitan area fixed effects and control for the racial/ethnic makeup of the population (share Black, share Latino, and share Asian), the share multifamily housing, as well as the percent change in median rent from 2009 to 2013. Full results reported in Appendix C Table C4.

**Table 4:** ML results Heckman selection model; dependent variable: log of multifamily permits 2014–2019.

Variables	1	2	3	4	5

Unbuilt capacity (log)	0.243**			0.127	
	(0.121)			(0.125)	
Prohibition index		−0.431**		−0.594**	
		(0.173)		(0.299)	
Process index			0.128**	0.236***	
			(0.063)	(0.076)	
WRLURI					−0.143
					(0.354)
Median rent, 2013 (log)	0.958	0.251	0.499	0.645	1.982
	(0.885)	(0.865)	(0.808)	(0.898)	(1.796)
Population (log)	1.514***	1.583***	1.595***	1.168***	1.042**
	(0.386)	(0.248)	(0.291)	(0.326)	(0.468)
Population density (log)	0.042	−0.448*	0.145	−0.061	−0.019
	(0.272)	(0.261)	(0.247)	(0.355)	(0.484)
Job accessibility (log)	−0.293	0.0519	−0.550***	−0.319	−0.355
	(0.196)	(0.195)	(0.192)	(0.223)	(0.354)
Multifamily housing (%)	3.566**	2.423**	3.275**	3.162***	1.531
	(1.537)	(1.029)	(1.274)	(1.213)	(1.705)
Constant	−19.92**	−12.01**	−14.60**	−12.03*	−16.96
	(7.954)	(5.490)	(6.420)	(6.140)	(12.340)
Observations	244	186	174	92	121
Lambda (Inverse Mills)	2.049	1.240	0.947	0.252	−2.206
	(1.656)	(1.636)	(1.044)	(1.077)	(2.314)

Notes: ***, ** and * indicate significance at the 0.01, 0.05, and 0.10 levels. Robust standard errors in parentheses. Models metropolitan area fixed effects and controls for the share Black, share Latino, share Asian, and the percent change in median rent from 2009 to 2013. Full results and selection model results reported in Appendix C Tables C5 and C6.

**Table 5:** OLS (Models 1–3) and Heckman twostep (Models 4–6) results; dependent variables: logged values of all permits and multifamily permits.

	Log permits 2014–2019			Log multifamily permits 2014–2019		
Variables	1	2	3	4	5	6
Unbuilt capacity (log)	−3.581***			−4.460*		
	(0.944)			(2.391)		
Prohibition index		0.707			2.053	
		(1.971)			(2.892)	
Process index			0.635			0.550
			(1.309)			(1.917)
Rent * Unbuilt capacity (log)	0.542***			0.649**		
	(0.129)			(0.329)		
Rent * Prohibition index		−0.112			−0.330	
		(0.257)			(0.375)	
Rent * Process index			−0.081			−0.051
			(0.181)			(0.261)
Median rent, 2013 (log)	−2.684***	0.519	0.760	−3.050	0.275	0.943
	(0.964)	(0.430)	(0.792)	(2.255)	(0.585)	(1.080)
Population (log)	0.856***	1.214***	1.214***	1.634**	1.548***	1.661***
	(0.095)	(0.073)	(0.099)	(0.732)	(0.410)	(0.452)
Population density (log)	−0.202*	−0.48***	−0.184	0.286	−0.213	0.359
	(0.122)	(0.154)	(0.174)	(0.476)	(0.236)	(0.251)
Job accessibility (log)	0.006	0.121	−0.205	−0.028	0.177	−0.154
	(0.079)	(0.110)	(0.146)	(0.274)	(0.173)	(0.199)
Constant	15.202**	−7.928**	−9.168	4.553	−13.85*	−22.47**
	(7.340)	(3.247)	(5.701)	(20.740)	(8.255)	(10.101)
Observations	404	225	229	244	186	174
R2	0.678	0.637	0.532			
Lambda (Inverse Mills)				3.378	0.885	1.211
				(3.446)	(1.589)	(1.456)

Notes: ***, ** and * indicate significance at the 0.01, 0.05, and 0.10 levels. Robust standard errors in parentheses. Models include metropolitan area fixed effects.

**Fig. C2.**
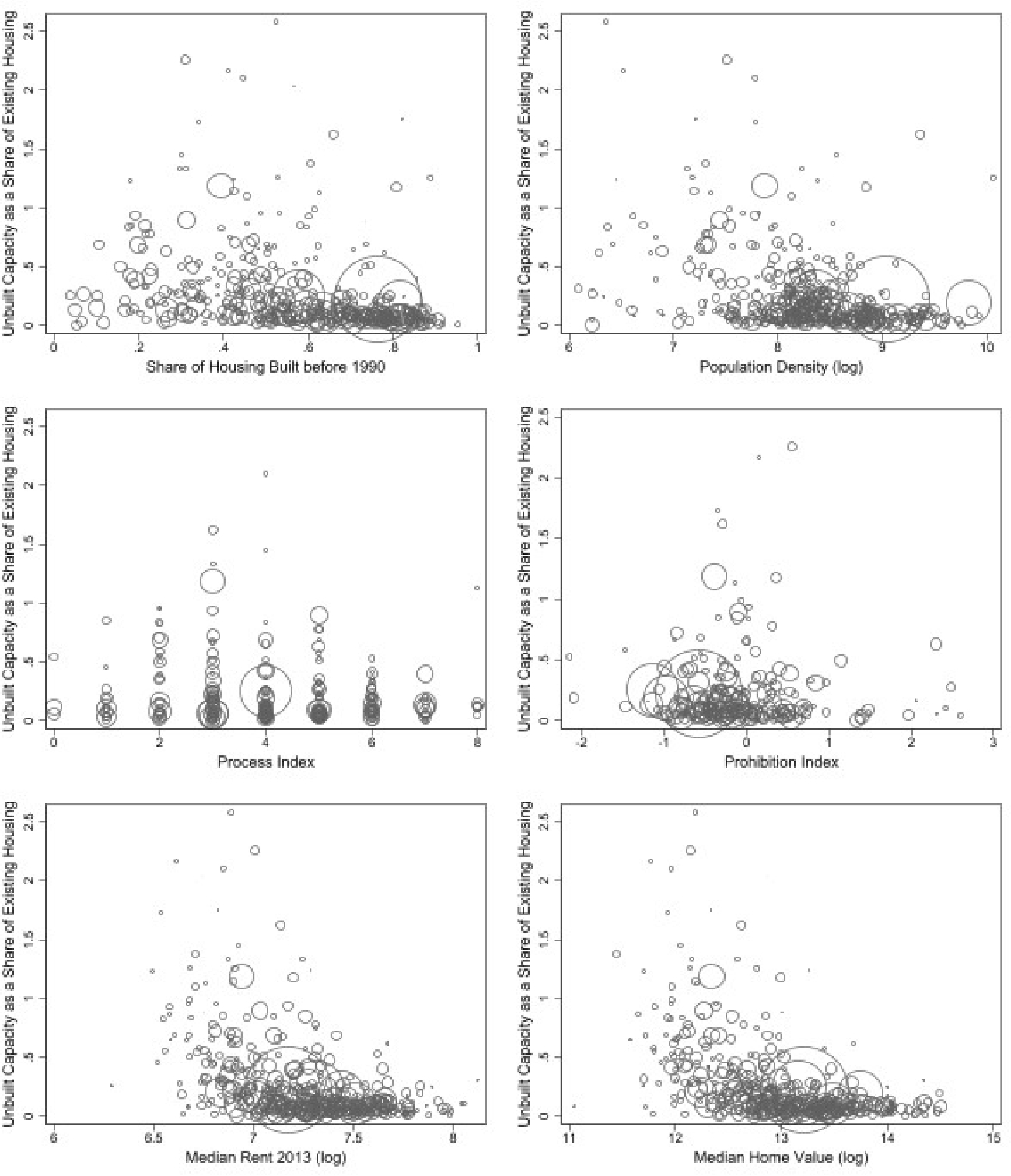
Scatterplots of unbuilt capacity with potential correlates: regulatory prohibitions, regulatory process, population density, rents, value, and the age of the housing stock.

## Data Availability

We provide a link to our unbuilt capacity data: www.lewis.ucla.edu/publications/housing-element

## Footnotes

1 Quigley and Rosenthal (2005) identified 50 papers in this literature. We identified another 25, which we summarize in Appendix A.

2 Appendix A shows that supply is the outcome in only 12 of the 80 papers we examined.

3 The literature tends to measure prices using purchase prices or home values, even though rents are arguably a better measure of housing’s consumption cost. None of the studies we reviewed since 2005 have used rents as an independent variable.

4 Surveys account for over 20 of the 80 papers we reviewed.

5 Some surveys, such as Jackson (2018) succeed in having planning directors or other high-level officials respond to them. Even these surveys, however, may not accurately capture the many regulations and processes that planning departments essentially outsource to sister agencies, such as a Building and Safety Department or a Department of Public Works.

6 For more discussion, see the comments on Lewis and Marantz (2019) in the *Journal of the American Planning Association* (volume 85 issue 4).

7 An additional factor under city control is the ease of entry for firms, which we cannot directly measure but which we assume is roughly the same across California municipalities.

8 Although as we have seen, planners do still err when reporting shares of land zoned multi- or single-family.

9 For a discussion of scheduled and discretionary exactions see Manville et al. (2023).

10 State guidelines use density as a proxy for household income. Income requirements thus require zoning at density thresholds, e.g. jurisdictions in metropolitan statistical areas must have sites developable at 30 units per acre to satisfy targets for low-income housing.

11 As we will discuss, not every city reports multifamily capacity. But every city *is* required to estimate total capacity, and virtually all do.

12 See the website of HCD for details on the HE guidelines: http://www.hcd.ca.gov/community-development/building-blocks/index.shtml

13 For example, the 2021 report for West Hollywood, a small city in Los Angeles County, is 28 pages of main prose with a 154-page technical appendix. The city hired a consulting firm, assigned full-time planners, and assembled a working group of residents to complete it, and the process took over a year. The typical land use survey, in contrast, is 14–30 pages long and usually filled out by a single person.

14 Observers sometimes characterize the RHNA process as requiring cities to “build” housing but the law only compels them to make room for housing in their plans.

15 The state began clamping down on strategic behavior midway through the 6^th^ RHNA cycle (Monkkonen et al 2023).

16 The RHNA process lends itself to such manipulation in part because it was designed in an era when almost all cities had abundant vacant land, and were thus expected to meet their targets by identifying vacant parcels (Baer, 2008).

17 Beverly Hills illustrates our reasoning here. The city is well known for behaving strategically in the RHNA process (Dillon, 2017). In 2019, the city protested its multifamily housing allocation and warned, in a letter to the regional government, that forcing it to add apartments would actually undermine affordability. The city reasoned that because of its land constraints, it could only add more multifamily housing if it demolished its existing rent controlled apartment buildings and redeveloped those sites at higher densities. This statement is absurd. Most of Beverly Hills’s residential land is flat terrain zoned for detached single-family housing, and the vast majority of these properties sell for well over $2 million. Nothing but the city’s zoning stops these sites from being redeveloped at higher densities. The city’s warning about apartment demolition is only true if it chooses not to rezone, and nothing, other than politics, stops it from rezoning.

18 At the risk of belaboring this point, the illegality of subdividing a single-family house into apartments almost by itself undermines the idea that “land constraints” are the binding obstacle to housing supply. Such subdivision can dramatically increase the number of units without changing the footprint of any existing buildings.

19 All but three of the municipalities that did not permit new multifamily housing do have *existing* multifamily housing. In fact, 22 % of the housing stock of the median municipality that did not permit any during this time period was multifamily housing, only slightly lower than the median municipality that did permit new multifamily (at 31 % of stock).

20 In Fig. C1, we present a graph of the distribution of unbuilt capacity as a share of existing housing across regions.

21 We include scatterplots showing the relationship between unbuilt capacity and these potential correlates in Fig. C2.

22 Palmdale, for example, is in the top 5 % of stringency as measured by the Prohibition Index (multifamily housing is not allowed in most of the city), but also top 5 % of unbuilt capacity (it has a lot of vacant parcels). Rancho Santa Margarita, in contrast, scores near the bottom on the Prohibition Index, but is by its own judgment almost completely built out. It reported only two units of unbuilt capacity in its housing element. Nearly 50 of the 250 cities for which we have regulation data score below average on regulatory stringency, above average in share multifamily, but have a below average level of unbuilt capacity.

23 Beverly Hills again provides an illustration. The city, which had 13,000 housing units and a median rent of $2043 in 2013, was given a RHNA allocation of two units and reported an unbuilt capacity of 732 (Dillon, 2017). In contrast, in the same year the City of Coachella, which had 15,000 housing units and a median rent of $897, was assigned over 5000 units and reported an unbuilt capacity of over 10,000.

24 Furth and Gonzalez’s (2019) analysis of this measure of regulation finds a substantial role played by outliers in a linear analysis. Unbuilt capacity is not sensitive to outliers in the same manner.

25 As a robustness check for this comparison, we ran the models using interaction terms with unbuilt capacity on the smaller sample size of places with data on prohibitions and process. In both cases the interactions are significant and the effect sizes are actually larger.

26 The 13 cities with high rents and high absolute capacity are Brentwood, Fremont, Hermosa Beach, Irvine, Milpitas, Newport Beach, San Jose, San Mateo, San Ramon, Santa Clara, Santa Clarita, and Sunnyvale. The five cities with high rents and high capacity relative to size are: Emeryville, Hercules, Hermosa Beach, La Habra Heights, and Milpitas.

## Appendix A

See Tables A1, A2

**Table A1:** Summary of empirical literature linking land use regulation to housing supply.

Author(s)	Year	Geography covered	Dependent variable	Regulation measure	Model type

Thorson (1997)	1997	Parcels in IL	Permits (log)	Specific: Agricultural downzoning	Stock-flow, 1979–1984 & 1985–1994
Skidmore and Peddle (1998)	1998	Cities in IL	Change in Units (using permits)	Specific: Development Impact Fees	Fixed effects panel
Levine (1999)	1999	Cities in CA	Change in Units (using Census data)	An index: Surveys of several areas	OLS, change 1980–1990
Mayer and Somerville (2000)	2000	U.S. metros	Permits (log)	Specific: Development or impact fees, delays	Panel (quarterly) 1985 to 1996
Quigley et al. (2004)	2004	Cities in CA	Change in Units (using permits)	An index: Surveys of several areas	OLS, 1990–2000
Zabel and Paterson (2006)	2006	Cities in CA	Single family permits	Specific: Critical habitat designation	Difference in difference 1990–2002
Glaeser and Ward (2009)	2009	Municipalities in Boston	Permits (log)	Specific: Lot sizes, wetlands by-laws, septic regulations, and subdivision rules	OLS, three cross sections
Schuetz (2009)	2009	Municipalities in MA	Permits (log)	Specific: Multifamily permitting rules	IV using historical characteristics
Kahn (2011)	2011	Cities in CA	Permits (log)	An index: Political ideology	Panel (annual) 2000 to 2008
Dempsey and Plantinga (2013)	2013	Cities in OR (parcels)	A plot being developed	Specific: Urban growth boundaries	Difference-in-difference
Jackson (2016)	2016	Cities in CA	Permits (log)	An index: Surveys of several areas	Panel, 1970–1995
Murray and Schuetz (2019)	2019	Cities in CA	Permits per 10,000 people	Specific: Maximum density, height, and % zoned multifamily	Tobit, change 2013–2018

**Table A2:** Summary of empirical literature linking land use regulation to housing prices (building on the summary in Quigley and Rosenthal (2005)).

Author(s)	Year	Geography covered	Dependent variable	Regulation measure	Model type

Quigley and Rosenthal (2005)	2005	Cities in CA	House price (1990 and 2000)	Index: Based on surveys	Hedonic model
Green et al. (2005)	2005	45 US metro areas	Supply elasticity	Index: Based on surveys	OLS
Ihlanfeldt (2007)	2007	Cities and counties in FL	House and land price (2000–2002)	Index: Based on surveys	Two stage least squares
Glaeser and Ward (2009)	2009	Cities & towns in Greater Boston	House price (2000 and 2005)	Index: Based on surveys	OLS
Saiz (2010)	2010	MSAs in the USA	Supply elasticity	Index: Based on surveys and a measure of developable land	Two stage least squares
Ball (2011)	2011	Southern England	Time to receive residential development approval	Specific: sites features, proposed buildings, local approval authorities, developers	OLS
Kahn et al. (2010)	2010	Parcels in CA	Housing units and house prices (2008)	Specific: Coastal boundary zone	Regression discontinuity
Zabel and Dalton (2011)	2011	Towns in MA	House prices (1987–2006)	Specific: Minimum lot size	OLS and difference-in-difference
Huang and Tang (2012)	2012	Cities in the US	House prices (2000 and 2009)	Index: Based on surveys	Fixed effects model
Kok et al. (2014)	2014	Cities in the San Francisco Bay Area	Land prices	Index: Based on surveys	OLS
Munneke et al. (2014)	2014	Housing near Brigham Young University	Housing prices	Specific: University policy limiting student housing location	Flexible hedonic model
Hilber and Vermeulen (2016)	2016	Planning authorities in England	Mixed- adjusted house price index	Specific: Refusal rate of large residential projects	Panel (1974 to 2008)
Jackson (2016)	2018	Cities and Counties in CA	Zillow Home Value Index (ZHVI)	Index: Based on surveys	Two-way fixed effects model
Gyourko and Krimmel (2021)	2021	CBSAs in the US	Land prices	Index: Based on surveys	Zoning tax estimates

## Appendix B. Components of the regulatory process index

The process index is a sum of eight binary variables created from questions in Jackson (2016). We coded variables 1 if they were above or below the median value in the direction of “more restrictive”. For example, in question 1 fewer meetings is more restrictive, and the median value is twice per month. So we coded cities with less than one or one meeting per month of their permit-granting entity are coded as 1 for this variable, and the rest are 0.

1. How many times a month (including special meetings) does your permit-granting entity typically meet to consider development applications? (Less than once per month, Once, Twice, Three times, Four times, More than four times a month)
2. Within how many days to you consider a typical single-family development application?
  (0–14, 15–29, 30–44, 45–59, 60 or more)
  Questions 3–5: For developments on land needing no rezoning, zoning amendment, bulk variance, etc., what is the typical time to secure preliminary plat/plan approval for the most common applications for the following types of development, starting from the time the application is deemed complete?
3. Single Family Detached Development
4. Townhouse residential development
5. Multifamily residential development
6. Apart from the body that grants preliminary plat/plan approval of the single-family detached development application, how many other boards and/or regulatory bodies immediate to the local jurisdiction must grant permission or preliminary approval before a typical residential development is approved in your jurisdiction?
  (None, One, Two or Three, Four or Five, More than Five)
7. Does your jurisdiction offer pre-application conferences, sketch/concept reviews, or similar measures designed to expedite or resolve conflicts about residential development approval? If so, how long do these last?
  (No, Yes: One meeting, Yes: Several meetings, Yes: But the number of meetings varies so much it is impossible to say)
8. Who is typically authorized to grant preliminary plat/plan approval (at time of vested rights) for single family detached development application?
  (No local approvals are required for subdivisions in this jurisdiction, Staff, Appointed or elected citizen board (planning board or commission), Elected legislative body)

## Appendix C

See Figs. C1–C3, C1–C9

**Table C1:** Pairwise correlations between measures of regulation and permits.

Variables	Unbuilt capacity (log)	Unbuilt multifamily capacity (log)	MATCHR	Process index	WRLURI	Permits 2014–2019 (log)

Unbuilt Multifamily Capacity (log)	0.91					
*N*	346					
MATCHR	−0.28	−0.30				
*N*	235	190				
Process Index	−0.10	−0.12	0.07			
*N*	236	193	144			
WRLURI	0.04	0.03	0.10	0.28		
*N*	150	121	98	100		
Permits 2014–2019 (log)	0.64	0.62	−0.22	0.02	0.23	
*N*	420	340	241	249	159	
Multifamily Permits 2014–2019 (log)	0.56	0.53	−0.33	0.15	0.23	0.84
*N*	310	252	196	186	127	322

**Table C2:** Data availability for metropolitan cities by California region.

Region	Total number of cities	Process index*	Prohibition index	WRLURI	Total unbuilt capacity	MF unbuilt capacity

Greater LA	191	109	99	73	182	138
SF Bay Area	108	54	67	33	96	78
San Diego	19	8	13	7	18	18
Sacramento	29	11	10	9	23	20
Monterey Bay	17	8	9	4	15	11
Fresno	16	8	5	5	15	13
Small Metros	81	33	25	29	65	57
Total cities	461	231	228	160	414	335

Notes: This table shows availability by city for each metropolitan region of California for data from four different data sources—three surveys (Jackson, 2016; Furth and Gonzalez, 2019 based on Mawhorter, 2019; Mawhorter and Reid, 2018; Gyourko et al., 2019) and unbuilt capacity estimates from cities’ housing elements.

**Table C3:** Distribution of the main regulatory and production variables.

	Percentile				
Variable	10th	25th	50th	75th	90th

Housing Element targets as a share of existing stock	0.01	0.03	0.05	0.12	0.22
Housing Element targets as a share of unbuilt capacity	0.07	0.22	0.43	0.77	1.00
Unbuilt capacity	191.00	580.00	1641.00	4514	10032.00
Unbuilt multifamily capacity	91.00	280.00	954.00	2352	5207.00
Unbuilt capacity as a share of existing housing units	0.03	0.06	0.13	0.32	0.72
Unbuilt multifamily capacity as a share of existing housing units	0.02	0.03	0.07	0.16	0.32
The MATCHR measure of regulatory prohibitions	−0.77	−0.47	−0.14	0.29	0.73
The Process Index	2.00	3.00	4.00	5.00	6.00
The WRLURI	−0.48	−0.06	0.70	1.39	2.01
All permits 2014–2019	14.00	73.00	271.00	903.00	2462.00
Multifamily permits 2014–2019	0.00	0.00	42.00	291.00	1020.00

**Table C4:** Full results for Table 3. OLS results, dependent variable: log of all permits 2014–2019.

Variables	1	2	3	4	5

Unbuilt capacity (log)	0.362***			0.333***	
	(0.075)			(0.119)	
Prohibition index		−0.101		−0.371**	
		(0.132)		(0.184)	
Process index			0.040	0.007	
			(0.048)	(0.069)	
WRLURI					0.074
					(0.135)
Median rent, 2013 (log)	0.906**	0.355	−0.430	0.642	−0.722
	(0.409)	(0.613)	(0.607)	(0.792)	(0.916)
Population (log)	0.905***	1.259***	1.277***	0.961***	1.260***
	(0.101)	(0.077)	(0.099)	(0.178)	(0.136)
Population density (log)	−0.157	−0.530***	−0.113	−0.473**	−0.027
	(0.134)	(0.160)	(0.183)	(0.221)	(0.267)
Job accessibility (log)	−0.072	0.063	−0.412***	−0.239	−0.227
	(0.091)	(0.126)	(0.153)	(0.171)	(0.217)
Multifamily housing (%)	−11.180***	−6.392	−0.270	−5.887	0.755
	(2.989)	(4.277)	(4.109)	(5.595)	(6.440)
Black population (%)	−3.423***	−2.766**	−3.137*	−3.697*	−2.265
	(1.156)	(1.389)	(1.727)	(2.074)	(3.131)
Asian population (%)	0.700	0.463	0.867	1.346*	−0.232
	(0.437)	(0.684)	(0.658)	(0.763)	(0.909)
Hispanic population (%)	−0.024	0.0481	−0.963	−0.148	−1.695*
	(0.413)	(0.595)	(0.636)	(0.837)	(0.883)
Change in rent 2009–2013 (%)	−0.183	−0.121	1.064	−0.722	0.130
	(0.476)	(0.734)	(0.938)	(1.682)	(1.240)
Constant	−11.180***	−6.392	−0.270	−5.887	0.755
	(2.989)	(4.277)	(4.109)	(5.595)	(6.440)
Observations	404	225	229	133	147
Pseudo R2	0.669	0.646	0.565	0.639	0.593

Notes: ***, ** and * indicate significance at the 0.01, 0.05, and 0.10 levels. Robust standard errors in parentheses. Models include metropolitan area fixed effects.

**Table C5:** Full results for Table 4. Heckman twostep model, dependent variable: log multifamily permits 2014–2019.

Variables	1	2	3	4	5

Unbuilt capacity (log)	0.243**			0.127	
	(0.121)			(0.125)	
Prohibition index		−0.431**		−0.594**	
		(0.173)		(0.299)	
Process index			0.128**	0.236***	
			(0.063)	(0.077)	
WRLURI					−0.143
					(0.354)
Median rent, 2013 (log)	0.958	0.251	0.499	0.645	1.982
	(0.885)	(0.865)	(0.808)	(0.898)	(1.796)
Population (log)	1.514***	1.583***	1.595***	1.168***	1.042**
	(0.386)	(0.248)	(0.291)	(0.326)	(0.468)
Population density (log)	0.042	−0.448*	0.145	−0.061	−0.019
	(0.272)	(0.261)	(0.247)	(0.355)	(0.484)
Job accessibility (log)	−0.293	0.052	−0.550***	−0.319	−0.355
	(0.196)	(0.195)	(0.192)	(0.223)	(0.354)
Multifamily housing (%)	3.566**	2.423**	3.275**	3.162***	1.531
	(1.537)	(1.029)	(1.274)	(1.213)	(1.705)
Black population (%)	−6.233	−4.408	−1.900	−3.040	4.761
	(3.991)	(3.581)	(3.041)	(4.716)	(7.115)
Asian population (%)	1.348	0.845	1.425	1.043	−1.385
	(1.146)	(1.023)	(0.885)	(1.096)	(2.137)
Hispanic population (%)	0.543	0.403	0.443	1.296	−0.223
	(0.890)	(0.779)	(0.810)	(0.917)	(1.652)
Change in rent 2009–2013 (%)	1.355	0.695	2.715*	−0.773	1.207
	(1.094)	(1.150)	(1.436)	(1.791)	(2.142)
Constant	−19.92**	−12.01**	−14.60**	−12.03*	−16.96
	(7.954)	(5.490)	(6.420)	(6.140)	(12.340)
Observations	334	228	231	124	148
Lamda (Inverse Mills)	2.049	1.240	0.947	0.252	−2.206
	(1.656)	(1.636)	(1.044)	(1.077)	(2.314)

Notes: ***, ** and * indicate significance at the 0.01, 0.05, and 0.10 levels. Robust standard errors in parentheses. Models include metropolitan area fixed effects.

**Table C6:** Selection model results for Table 4. Dependent variable log of multifamily permits 2014–2019.

Variables	1	2	3	4	5

Unbuilt capacity (log)	0.008			−0.032	
	(0.069)			(0.129)	
Prohibition index		−0.033		−0.706**	
		(0.148)		(0.295)	
Process index			0.002	−0.029	
			(0.065)	(0.123)	
WRLURI					0.320*
					(0.175)
Median rent, 2013 (log)	0.140	−0.588	−0.184	−0.323	−0.593
	(0.421)	(0.619)	(0.575)	(0.919)	(0.783)
Population (log)	0.468***	0.416***	0.733***	0.731***	0.485***
	(0.112)	(0.118)	(0.137)	(0.227)	(0.168)
Population density (log)	0.046	−0.199	0.204	−0.611*	0.109
	(0.156)	(0.207)	(0.193)	(0.341)	(0.271)
Job accessibility (log)	−0.084	0.162	−0.171	0.009	−0.112
	(0.108)	(0.148)	(0.147)	(0.225)	(0.194)
Multifamily housing (%)	1.748***	0.901	2.290**	1.677	0.184
	(0.579)	(0.873)	(0.929)	(1.196)	(0.925)
Black population (%)	−3.698*	−4.746**	−4.837**	−9.811***	−4.875
	(1.925)	(1.959)	(2.290)	(3.602)	(3.770)
Asian population (%)	0.414	−0.812	0.445	1.247	1.651
	(0.770)	(0.935)	(0.942)	(1.435)	(1.368)
Hispanic population (%)	−0.122	−0.453	−0.445	−0.402	0.928
	(0.471)	(0.640)	(0.596)	(0.931)	(0.796)
Change in rent 2009–2013 (%)	0.250	0.836	−0.927	−0.141	−0.543
	(0.612)	(0.900)	(0.982)	(1.722)	(1.201)
Constant	−5.380*	0.825	−5.954	0.127	−0.104
	(2.934)	(4.251)	(3.724)	(6.127)	(5.448)
Observations	334	228	231	124	148

Notes: ***, ** and * indicate significance at the 0.01, 0.05, and 0.10 levels. Robust standard errors in parentheses. Models include metropolitan area fixed effects.

**Table C7:** OLS results; dependent variable is permits 2014–2019 as a share of housing units in 2013.

Variables	1	2	3	4	5

Unbuilt capacity (log)	1.716***			1.477***	
	(0.333)			(0.421)	
Prohibition index		−1.534**		−3.093***	
		(0.718)		(1.043)	
Process index			0.369	0.025	
			(0.272)	(0.286)	
WRLURI					−0.769
					(0.845)
Median rent, 2013 (log)	4.100**	5.266*	−1.271	6.926	−4.920
	(1.870)	(2.794)	(3.516)	(4.398)	(4.559)
Population (log)	−0.984**	0.867***	0.338	−0.821	0.380
	(0.445)	(0.317)	(0.589)	(0.684)	(0.676)
Population density (log)	−0.291	−2.654***	−1.985*	−4.090***	−1.540
	(0.381)	(0.820)	(1.072)	(1.152)	(1.655)
Job accessibility (log)	−0.107	−0.410	−0.380	−0.839	0.353
	(0.427)	(0.480)	(1.184)	(0.787)	(1.709)
Multifamily housing (%)	0.0216	−0.626	1.267	2.365	−0.972
	(2.247)	(3.486)	(4.248)	(3.759)	(5.403)
Black population (%)	−10.41**	−3.150	−8.822	−9.546	−16.22
	(4.161)	(4.963)	(6.898)	(9.220)	(14.99)
Asian population (%)	1.626	5.895*	3.083	12.270***	−6.135
	(2.587)	(3.421)	(4.263)	(4.066)	(5.713)
Hispanic population (%)	1.990	4.485*	0.842	7.510*	−6.657
	(1.919)	(2.423)	(3.338)	(3.871)	(4.441)
Change in rent 2009–2013 (%)	2.400	0.687	14.74*	5.863	18.47**
	(2.986)	(3.123)	(7.813)	(9.696)	(9.184)
Constant	−25.55*	−19.72	23.91	−13.89	46.05
	(13.45)	(18.79)	(24.08)	(30.74)	(33.64)
Observations	412	227	231	135	148
	0.249	0.258	0.221	0.484	0.222

Notes: ***, ** and * indicate significance at the 0.01, 0.05, and 0.10 levels. Robust standard errors in parentheses. Models include metropolitan area fixed effects.

**Table C8:** OLS results; dependent variable is multifamily permits 2014–2019 as a share of housing units in 2013.

Variables	1	2	3	4	5

Unbuilt capacity (log)	0.343***			0.095	
	(0.111)			(0.176)	
Prohibition index		−0.325		−0.995*	
		(0.230)		(0.542)	
Process index			0.202*	0.196	
			(0.106)	(0.144)	
WRLURI					0.090
					(0.249)
Median rent, 2013 (log)	1.094	1.543	−0.277	3.305**	−0.970
	(1.071)	(1.221)	(2.007)	(1.537)	(2.173)
Population (log)	0.109	0.770***	0.623***	0.597	0.175
	(0.193)	(0.148)	(0.180)	(0.363)	(0.312)
Population density (log)	0.0194	−0.766**	−0.0921	−1.102**	0.289
	(0.374)	(0.335)	(0.461)	(0.509)	(0.443)
Job accessibility (log)	−0.225	−0.037	−0.633**	−0.336	−0.467
	(0.172)	(0.203)	(0.316)	(0.381)	(0.295)
Multifamily housing (%)	3.041**	4.292***	3.526*	4.941**	0.612
	(1.357)	(1.501)	(1.830)	(2.173)	(1.926)
Black population (%)	−7.413***	−5.230**	−5.413	−8.802*	−9.526
	(2.519)	(2.270)	(3.422)	(5.068)	(6.132)
Asian population (%)	1.338	3.243	3.127	4.484**	0.415
	(1.473)	(2.274)	(2.226)	(2.222)	(2.137)
Hispanic population (%)	0.712	1.792*	0.566	3.679**	−0.858
	(1.227)	(1.034)	(1.856)	(1.576)	(2.030)
Change in rent 2009–2013 (%)	3.482	1.407	8.515	−2.710	8.878
	(2.573)	(1.555)	(6.217)	(2.369)	(5.854)
Constant	−10.01	−13.68*	−1.004	−21.23**	6.675
	(7.141)	(7.530)	(13.08)	(9.585)	(16.55)
Observations	333	227	231	111	148
	0.241	0.350	0.274	0.389	0.364

Notes: ***, ** and * indicate significance at the 0.01, 0.05, and 0.10 levels. Robust standard errors in parentheses. Models include metropolitan area fixed effects.

**Table C9:** Robustness check using housing prices; OLS results dependent variable: log of all permits and log multifamily permits with interaction terms.

	All permits 2014–2019			Multifamily permits 2014–2019		
Variables	1	2	3	1	2	3

Unbuilt capacity (log)	−2.473***			−3.415*		
	(0.836)			(2.001)		
Prohibition index		−0.129			1.763	
		(2.122)			(5.820)	
Process index			0.845			−0.147
			(0.974)			(1.457)
Value * Unbuilt capacity (log)	0.219***			0.284*		
	(0.064)			(0.154)		
Value * Prohibition index		−0.001			−0.184	
		(0.154)			(0.434)	
Value * Process index			−0.062			0.021
			(0.075)			(0.111)
Median housing value, 2013 (log)	−0.917*	0.240	0.706**	−1.184	1.712***	0.291
	(0.475)	(0.219)	(0.358)	(1.225)	(0.628)	(0.547)
Population (log)	0.882***	1.233***	1.255***	1.596***	−0.432	1.758***
	(0.099)	(0.078)	(0.104)	(0.619)	(0.528)	0.391)
Population density (log)	−0.211*	−0.486***	−0.149	0.275	0.229	0.326
	(0.117)	(0.151)	(0.164)	(0.456)	(0.336)	(0.268)
Job accessibility (log)	−0.066	0.099	−0.311*	−0.183	0.241	−0.257
	(0.087)	(0.123)	(0.164)	(0.291)	(0.697)	(0.205)
Constant	8.044	−7.174**	−12.60***	−0.012	−15.800	−18.71**
	(6.502)	(2.861)	(4.743)	(21.840)	(14.000)	(9.507)
Observations	412	227	231	333	227	231
R2	0.67	0.64	0.54			
Lambda (Inverse Mills)				2.927	2.927	1.640
				(2.815)	−4.717	(1.428)

Notes: ***, ** and * indicate significance at the 0.01, 0.05, and 0.10 levels. Robust standard errors in parentheses. Models include metropolitan area fixed effects.
